# Constructing Cell-Free Expression Systems for Low-Cost Access

**DOI:** 10.1021/acssynbio.1c00342

**Published:** 2022-03-08

**Authors:** Fernando Guzman-Chavez, Anibal Arce, Abhinav Adhikari, Sandra Vadhin, Jose Antonio Pedroza-Garcia, Chiara Gandini, Jim W. Ajioka, Jenny Molloy, Sobeida Sanchez-Nieto, Jeffrey D. Varner, Fernan Federici, Jim Haseloff

**Affiliations:** † Department of Plant Sciences, University of Cambridge, CB2 3EA Cambridge, U.K.; ‡ ANID − Millennium Institute for Integrative Biology (iBio), FONDAP Center for Genome Regulation, Institute for Biological and Medical Engineering, Schools of Engineering, Medicine and Biological Sciences, Pontificia Universidad Católica de Chile, Santiago 8330005, Chile; § Robert Frederick Smith School of Chemical and Biomolecular Engineering, Cornell University, Ithaca, New York 14853, United States; ∥ Department of Biochemistry, Faculty of Chemistry, National Autonomous University of Mexico (UNAM), 04510 Mexico City, Mexico; ⊥ Department of Chemical Engineering and Biotechnology, University of Cambridge, CB3 0FD Cambridge, U.K.; # Department of Pathology, University of Cambridge, Tennis Court Road, CB2 1QP Cambridge, U.K.

**Keywords:** cell-free protein synthesis, lyophilization, NTPs, lactose, low-cost, maltodextrin

## Abstract

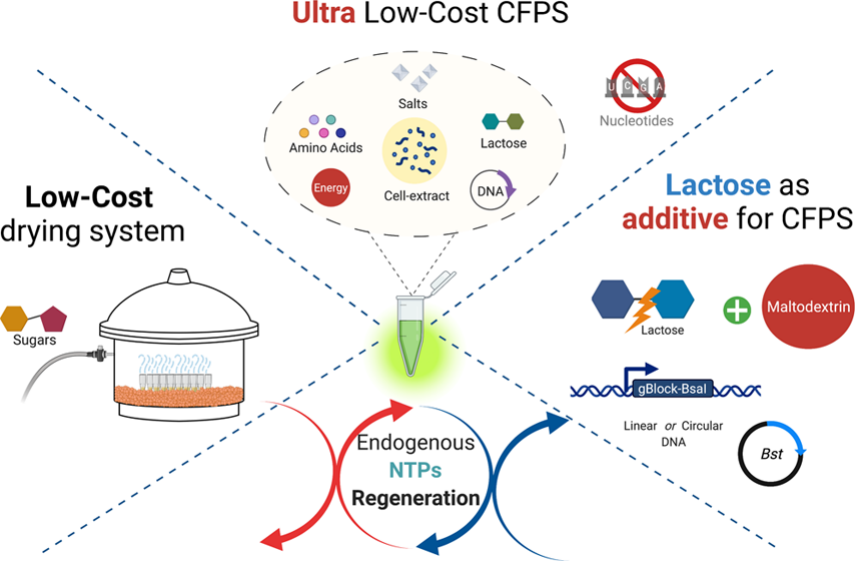

Cell-free systems for gene expression have gained attention as platforms for the facile study of genetic circuits and as highly effective tools for teaching. Despite recent progress, the technology remains inaccessible for many in low- and middle-income countries due to the expensive reagents required for its manufacturing, as well as specialized equipment required for distribution and storage. To address these challenges, we deconstructed processes required for cell-free mixture preparation and developed a set of alternative low-cost strategies for easy production and sharing of extracts. First, we explored the stability of cell-free reactions dried through a low-cost device based on silica beads, as an alternative to commercial automated freeze dryers. Second, we report the positive effect of lactose as an additive for increasing protein synthesis in maltodextrin-based cell-free reactions using either circular or linear DNA templates. The modifications were used to produce active amounts of two high-value reagents: the isothermal polymerase *Bst* and the restriction enzyme *Bsa*I. Third, we demonstrated the endogenous regeneration of nucleoside triphosphates and synthesis of pyruvate in cell-free systems (CFSs) based on phosphoenol pyruvate (PEP) and maltodextrin (MDX). We exploited this novel finding to demonstrate the use of a cell-free mixture completely free of any exogenous nucleotide triphosphates (NTPs) to generate high yields of sfGFP expression. Together, these modifications can produce desiccated extracts that are 203–424-fold cheaper than commercial versions. These improvements will facilitate wider use of CFS for research and education purposes.

## Introduction

Since the discovery of the functional relationship between mRNA and protein by Nirenberg and Matthaei in the early 1960s,^1^ cell-free systems (CFSs) have become powerful tools with broad applications now in synthetic biology, ranging from the study of artificial gene circuits and synthetic cells to protein production.^2−4^ The technology relies on *in vitro* transcription–translation systems that employ cell extracts as a source of ribosomes and auxiliary transcriptional factors,^5^ or reconstitution of purified cell components in the case of the PURE system.^6,7^ In addition to transcription–translation machinery, CFS requires an adequate supply of key elements such as amino acids, crowding reagents, salts, nucleotide triphosphates (NTPs), homeostatic environment, and an ATP regeneration system.^8^ There have been numerous reports of improvements in the way extracts are generated and fed. Several approaches have been used to find a better balance between protein yields and reagent costs, where nucleoside triphosphate and energy source represent more than 50% of the total cost of reactions.^9^ Energy substrates generally contain high-energy phosphate bonds, generating the ATP, necessary to carry out protein synthesis and other metabolic processes^10^ through simple phosphorylation reactions. However, phosphate donors such as phosphoenol pyruvate (PEP), creatine phosphate (CP), 3-phosphoglycerate (3-PGA), and fructose 1,6-bisphosphate are relatively expensive and require cold chain during storage and distribution, limiting the adoption of these cell-free technologies in low-resource settings or at a larger scale.^11^ To address this issue, ATP regeneration systems based on multienzyme reaction cascades associated with glycolysis and oxidative phosphorylation metabolism have been implemented.^12−14^ An example of this kind of energy source is maltodextrin (MDX), which also enhances protein production by limiting the production of excess phosphate levels in the reaction.^15^ Due to its low cost and high efficiency, the maltodextrin system was adopted and improved by Caschera et al. (2015), coupled with hexametaphosphate (HMP), as a phosphate donor to stimulate glycolysis.^16^ However, this ATP regeneration system has not been widely adopted as an energy source in CFS despite the fact that it has proven its efficiency for characterizing toehold sensors in low-cost contexts.^17^

So far, most efforts to reduce the costs of production and implementation of CFS have focused on alternative ATP regeneration systems, choice of reagents, and the optimization of working concentrations,^11,18,19^ leaving aside other critical aspects. For example, CFSs have gained attention in diagnostics due to their capacity to be lyophilized as pellets or in paper matrices, permitting the expression of a synthetic gene network in a point-of-care assay under contained conditions.^20,21^ Applications include the detection of Zika virus by coupling cell-free technology with isothermal RNA amplification and a toehold switch^22^ and the measurement of water contaminants through the RNA output sensors activated by ligand induction (ROSALIND) system.^23^ These advances have enabled the fabrication of low-cost, rapid diagnostics, but some remaining steps, such as the lyophilization of cell-free reaction components that are required for storage and distribution, still rely on access to expensive equipment.

To improve access to this technology, we have developed three novel approaches to reducing cost. First, we evaluated the capacity of silica beads coupled with a low vacuum to dry cell-free components based on two independent energy sources: PEP and MDX, using sugars as lyoprotectant agents to stabilize the mixtures. This is the first demonstration of the use of maltodextrin in a lyophilized mixture. These conditions allowed us to maintain 45 and 75% of protein synthesis capacity after 2 weeks of storage at room temperature in mixtures based on PEP and MDX, respectively, compared to fresh preparations.

Second, we found that the addition of lactose enhanced cell-free reactions, seeing a 188% increase in protein yield when maltodextrin was used as an energy source, giving comparable yields to those obtained with PEP. On this basis, we developed a modified version of the MDX cell-free formulation used it to produce active protein reagents: *Bst* DNA polymerase (commonly used in LAMP assays) using plasmid as template and the type IIS restriction enzyme *Bsa*I using linear DNA as a template. We developed a technique for the efficient use of linear DNA templates in the absence of stabilizers such as GamS or Chi DNA.^24,25^

Third, we found that endogenous biosynthesis of nucleoside mono- and triphosphate remains active in CFS reactions based on PEP. Thus, reactions could be composed without the addition of NTPs or NMPs^11^ in the mixture, contrary to normal practice. This allowed us to develop an ultra-low-cost cell-free system capable of producing 16.9 μM sfGFP using just cell extract, PEP, amino acids, salts, and lactose. This is likely to be the forerunner of a new class of cell-free expression systems that further employ closed biochemical circuits to regenerate essential reactants and to lower costs. The combined improvements described in this work facilitate the remote production of useful protein reagents and reduce the dependence on expensive equipment and supplies.

## Results and Discussion

### Drying Cell-Free Reactants Using a Low-Cost Protocol with Sugar Protectants

**Figure 1:**
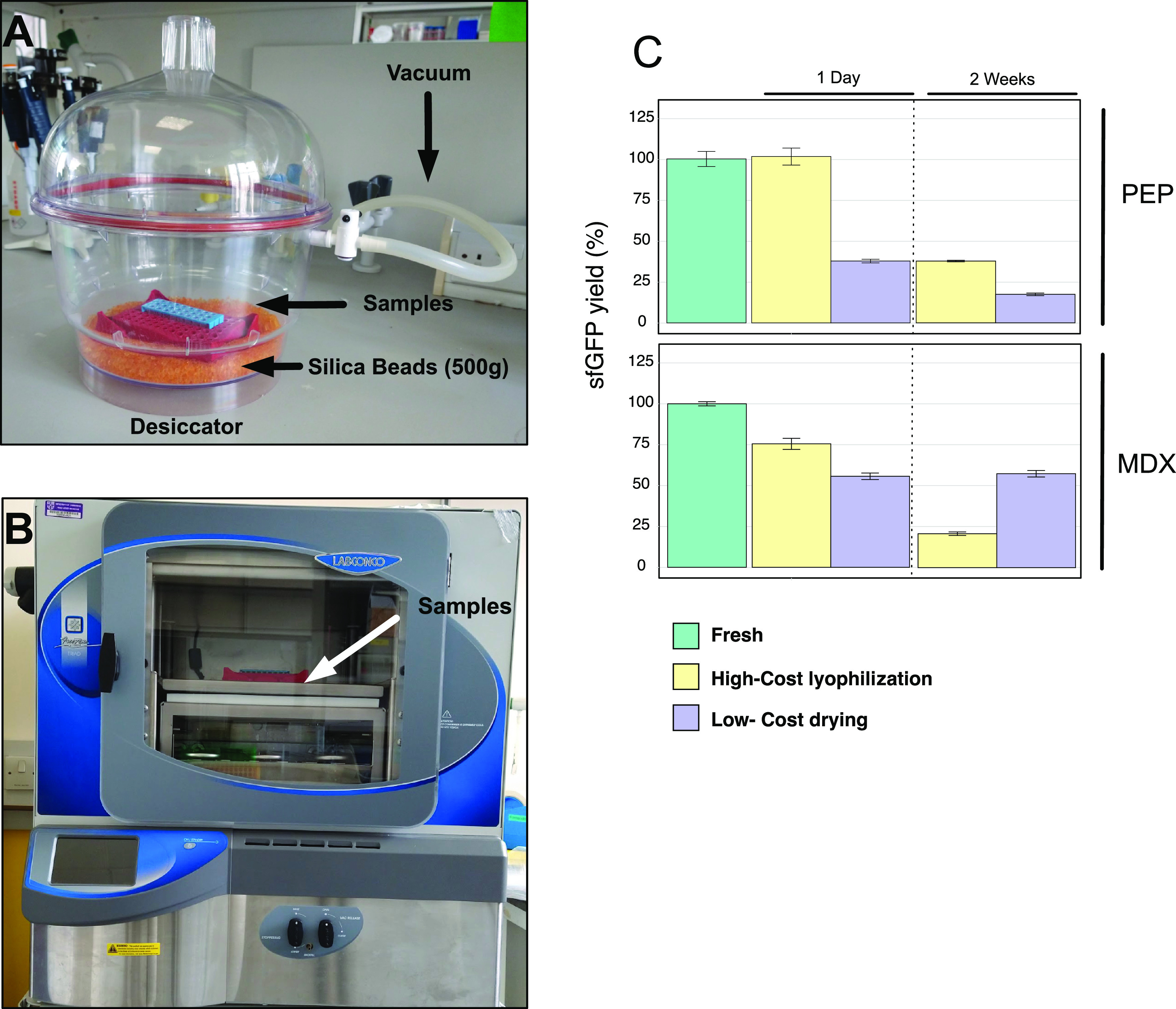
Drying systems to preserve active cell-free extracts. (A) Low-cost drying: low-vacuum, room-temperature drying apparatus using silica beads. (B) High-cost lyophilization: commercial, computer-controlled freeze dryer for lyophilization (FreeZone Triad Benchtop Freeze Dry System -Labconco). (C) Percentage of recovery after 1 day and 2 weeks of storage at room temperature in a sample. Cell-free reactions based on PEP (top panel) or MDX (bottom panel) as energy sources. Plasmid psfGFP was used as a DNA template. Percentage of recovery was calculated relative to the RFU value obtained from fresh extracts with the respective energy sources (PEP or MX). Cell-free reactions were incubated at 29 °C for 15 h. Error bars represent standard error over 12 technical measurements.

In recent years, cell-free systems have gained attention in the diagnostics field due to their versatility over traditional methods, particularly in high-throughput approaches using small-volume reactions and distribution to the point of care in a lyophilized form.^20,23,26^ However, the use of high-cost equipment required to lyophilize cell-free extracts remains a limitation for the local production of these diagnostic reactions. To overcome this challenge, we tested the use of a low-cost alternative (Figure 1A) to high-tech freeze dryers (Figure 1B) in two cell-free formulations based on different energy regeneration systems (Tables S1–S4). We observed that when samples using PEP as energy source were frozen and lyophilized without the addition of lyoprotectant in a computer-controlled commercial device (Table S4), they did not show a reduction in protein production 1 day after lyophilization (Figure 1C, top panel). However, only 31% of the initial activity remained after 2 weeks of storage at room temperature (23 °C). We also tested a low-cost drying device, where samples were simply kept overnight under low vacuum in a desiccator containing dry silica gel. When this device was used, the recovery was around 30% after 1 day and 20% after 2 weeks for the PEP-energized reactions (Figure 1C, top panel). Despite this drop in the recovery, the samples still showed high absolute levels of protein synthesis activity since yields for fresh samples were in the range of 40 μM of sfGFP. Longer-term storage of all dried samples was maintained in light-shielded, vacuum-bagged containers with desiccant in an argon-flushed, anoxic environment.

**Figure 2:**
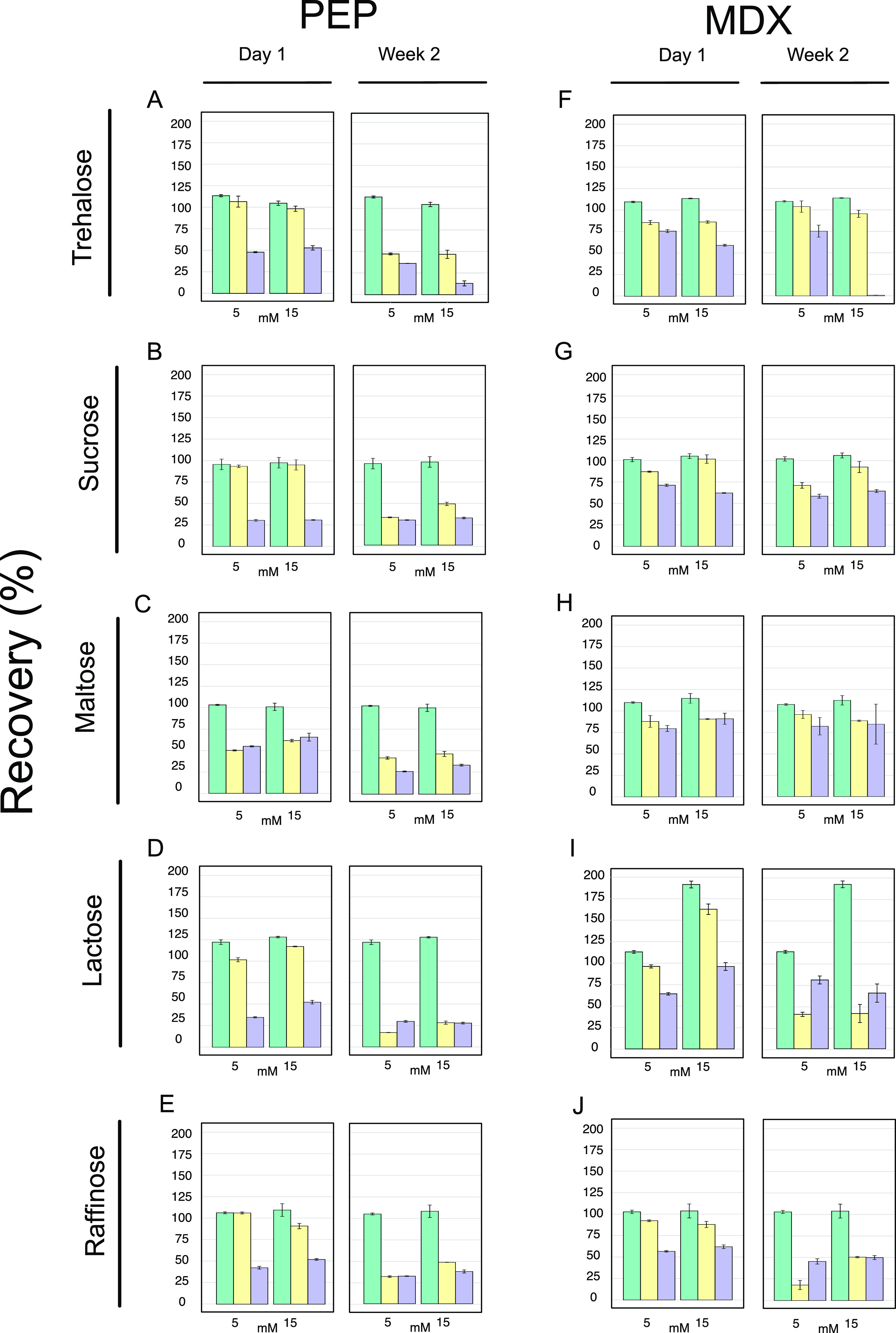

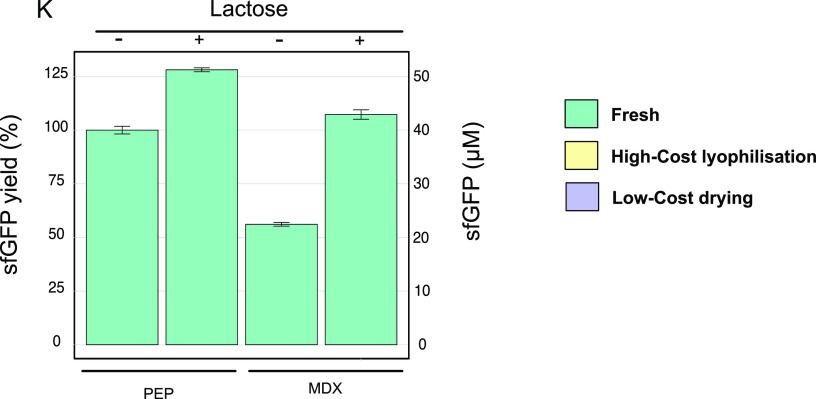
Lyoprotectant effects of five sugars individually added to the two cell-free formulations. CFPS based on PEP (A–E) and MDX (F–J) and dehydrated either by high-cost lyophilization or by the low-cost drying method. Samples were dried and stored at room temperature for 1 day and 2 weeks. Cell-free reactions were rehydrated and incubated at 29 °C for 15 h. The final concentrations of additives in the reactions are indicated on the horizontal axes. The percentage of the recovered protein production was calculated relative to that seen in fresh, additive-free reactions with the energy sources PEP or MDX. Error bars represent standard error over three technical measurements. (K) Effects of lactose on protein yields in fresh cell-free reactions based on PEP or MDX as energy sources. Samples were supplemented with lactose, 11.2 mM (PEP mixture; Tables S2 and S5) and 13.7 mM (MDX mixture; Tables S1 and S5) as indicated. Cell-free reactions for the production of a green fluorescent protein were incubated at 29 °C for 15 h using psfGFP as a DNA template. Yields were calculated relative to fluorescence values seen in PEP-formulated cell-free reactions in the absence of lactose. Error bars represent standard error over three technical measurements.

In contrast, cell-free reactions having maltodextrin as an energy source showed a better recovery after 2 weeks (50%) when simply dried over silica, compared to when the commercial freeze dryer was used (20%). Interestingly, the silica-dried samples showed the same level of stability from day 1 until 2 weeks later after storage of the dry reactions at room temperature (Figure 1C, bottom panel). This level of protection may be due to the presence of maltodextrin and PEG-8000^27,28^ in the reaction mixes. However, this does not explain the lower stability of samples dried by lyophilization. A possible explanation for this effect is the formation of ice crystals during the slow-freezing step before the lyophilization, which can cause structural damage in the cellular constituents present in the reaction mixture.^29^ Given that the PEP and MDX formulations (Tables S3 and S4) did not show a consistent difference in stability after drying or lyophilization, we evaluated the stabilizing and lyoprotectant properties of different sugars. Sugars are thought to act as water substitutes against dehydration through hydrogen bond interactions with dehydrated proteins,^30−33^ contributing to the stabilization of preferred protein conformations. Five sugars (trehalose, maltose, lactose, sucrose, and raffinose) at a range concentration from 0 to 120 mM (Table S5) were tested as protectants and compared to fresh cell-free reactions, evaluating the percentage of recovered activity at 1 day and 2 weeks after lyophilization (Figures 2A–J and S2A–J; duplicate reactions are described in Figure S1). It has been previously shown that trehalose can be an effective lyoprotectant in cell extracts.^34,35^ However, for PEP-containing extracts, we observed that sucrose and raffinose were the most effective stabilizers in both drying systems, showing activities of 75 and 45% after lyophilization or silica drying, respectively (Figure S2B,E). These yields were obtained by adjusting the sugar concentration in the reaction mixtures, reaching a maximum level of protection at 120 mM (Figure S2). In contrast, the rest of the sugars (maltose, lactose, trehalose) exhibited a negative effect as the concentration increased (Figure S2). In part, this may be due to molar ratios of stabilizer and protein.^36^ In the case of trehalose, previous studies suggested that a high concentration of this disaccharide can inhibit protein expression due to its high affinity for water molecules^35,37^ and consequent displacement of water from biomolecules. This might explain the low percentage of activity observed in samples dosed with this sugar (Figure S2A,F). Positive effects were seen when sugars were added as protectants along with maltodextrin in both drying processes, more pronounced when using the low-cost silica-drying device. The best results (around 75% of the original activity) were obtained when 5 mM trehalose, maltose, or lactose was added to the reaction mix (Figure 2F,H,I and Table S3), while the optimal concentration for sucrose was 5–15 mM (Figure 2G). The only two conditions that showed a high level of stabilization after lyophilization were the addition of maltose at low concentrations (5 and 15 mM) and sucrose at all of the tested concentrations (Figures 2G,H and S2G). Using trehalose, sucrose, and maltose (Figure 2F–H), the protein yields were the same after 1 day and 2 weeks of dry storage. On the basis of these observations, we decided to add sucrose as a protectant in reaction mixtures based on either PEP (120 mM; Table S4) or maltodextrin (15 mM; Table S3) for both lyophilization and silica-drying procedures due to the positive effect shown by this sugar under a wide range of conditions. This allowed us to use the cheapest sugar (USD $0.0066 per gram of sucrose) to minimize cost. Next, we sought to demonstrate the effectiveness of the approach by sending dried samples based on MDX as an energy source and protected with 15 mM sucrose for testing in Mexico and Chile (Figure S3A,B). The reaction mixtures were stable 2 weeks after drying, including transatlantic shipping and delays due to customs services in each country. In addition, the same batch of reactants was also successfully evaluated after 3 months (Figure S3C) in the United Kingdom along with the protective effect of sucrose at different concentrations in the MDX formulation (Figure S3D and Tables S3 and S5), showing up to % 60 recovery compared to fresh samples and demonstrating the robustness of our system in terms of stability and cost (Figure S8C). In summary, silica-based drying provides a new low-cost alternative to lyophilization for drying cell-free reactions, with potential use in diagnostic and education, using a cheap energy source of energy (MDX) and sucrose as a protective agent, which allows worldwide shipping and storage of reactants at room temperature.

### Lactose Enhances Cell-Free Reaction Yields

We observed that some sugars stimulated protein production in our cell-free expression systems, and we decided to evaluate their properties more systematically as additives. Sugars have been used as secondary energy sources in cell-free reactions to enhance protein yields.^9,10,38−40^ Recently, Moore et al.^41^ demonstrated that the combined use of 3-phosphoglyceric acid (3-PGA), with glucose-6-phosphate (G6P) as a secondary energy source, improved protein production in *Streptomyces* cell-free reactions 6-fold. Similarly, a beneficial effect on protein yields was observed when *Escherichia coli* cell extracts were supplemented with 30 mM d-ribose in a system based on MDX.^42^ Further, lyophilized *E. coli* cell-free extracts stored at −80 °C and rehydrated after 2 weeks showed increased activity if prepared with maltose, trehalose, or lactose, with PEP as the main energy force, suggesting that sugars were useful additives in cell-free reactions.^34^ These observations were consistent with our results with fresh samples, as an enhancement in protein production was seen, when PEP or MDX was used as the main source energy and relevant sugars were added (Figure 2 and Tables S5 and S6). Samples supplemented with trehalose recorded a maximum peak of expression (125%) at 3.7 mM concentration (Table S5), while in cell-free reactions containing maltose and lactose, the highest productivity was 112.5 and 128%, respectively, at concentrations of 11.2 mM (Figure 2 and Table S5). For the cell-free reactions based on maltodextrin, those supplemented with trehalose and maltose (Figure 2F,H) showed a 112.5% activity at an 11.2 mM concentration; however, it should be noted that the protein yields obtained using MDX, in general, represent about 50% of those achieved with PEP (Figure 2K). Surprisingly, the addition of 13.7 mM lactose boosted protein production to 188% (Figure 2I), allowing protein yields equal to those obtained with PEP (Figure 2K). A typical cell-free reaction uses simple substrate-level phosphorylation reactions to regenerate ATP using substrates with high-energy phosphate bonds such as PEP, acetate phosphate, glucose-6-phosphate (G6P), 3-phosphoglycerate, creatine phosphate (CP), or acetyl phosphate (AP).^9,10^ However, the consumption of these compounds contributes to increased inorganic phosphate in the medium, which can eventually result in the sequestration of free magnesium ions.^43^ Protein synthesis can be inhibited due to the lack of these ions, which are needed for essential reactions such as nucleoside triphosphate synthesis and protein translation.^44,45^ An alternative, which avoids phosphate accumulation, is the use of MDX as a substrate for ATP regeneration. MDX is slowly metabolized in the cell-free mix and contributes to oxidative phosphorylation reactions, which recycle inorganic phosphate coming from other metabolic processes and from the specific phosphate donor (HMP) added in the reaction mixture. Consequently, phosphate accumulation is reduced, fluctuations in pH are lower, and levels of ATP can be maintained for protein production.^15,16,39^ This may help explain the observed beneficial impact of lactose on protein synthesis observed when MDX is used instead of PEP to energize reactions (Figure 2K). We speculate that lactose is consumed by β-galactosidase (induced by IPTG during cell-extract preparation) present in the cell extracts,^21^ producing glucose as a secondary carbon source.

To further evaluate if lactose also acts as an enhancer in dried samples containing MDX in their formulation, lyophilized and silica-dried reactions were made with 15 mM of sucrose (Table S3) or with a mixture of 15 mM sucrose and 15 mM lactose as protectants and were rehydrated with 13.7 mM lactose or water, respectively, after 2 weeks of storage at room temperature. In contrast to earlier results in fresh samples supplemented with lactose (Figure 2I), we only observed a slight improvement in the cell-free reactions dried using the low-cost protocol (Figure S4). These results indicate that the enhancement due to lactose as an additive is only preserved in fresh cell-free reactions and not in the rehydrated samples and effects on protein stability.^46^

**Figure 3:**
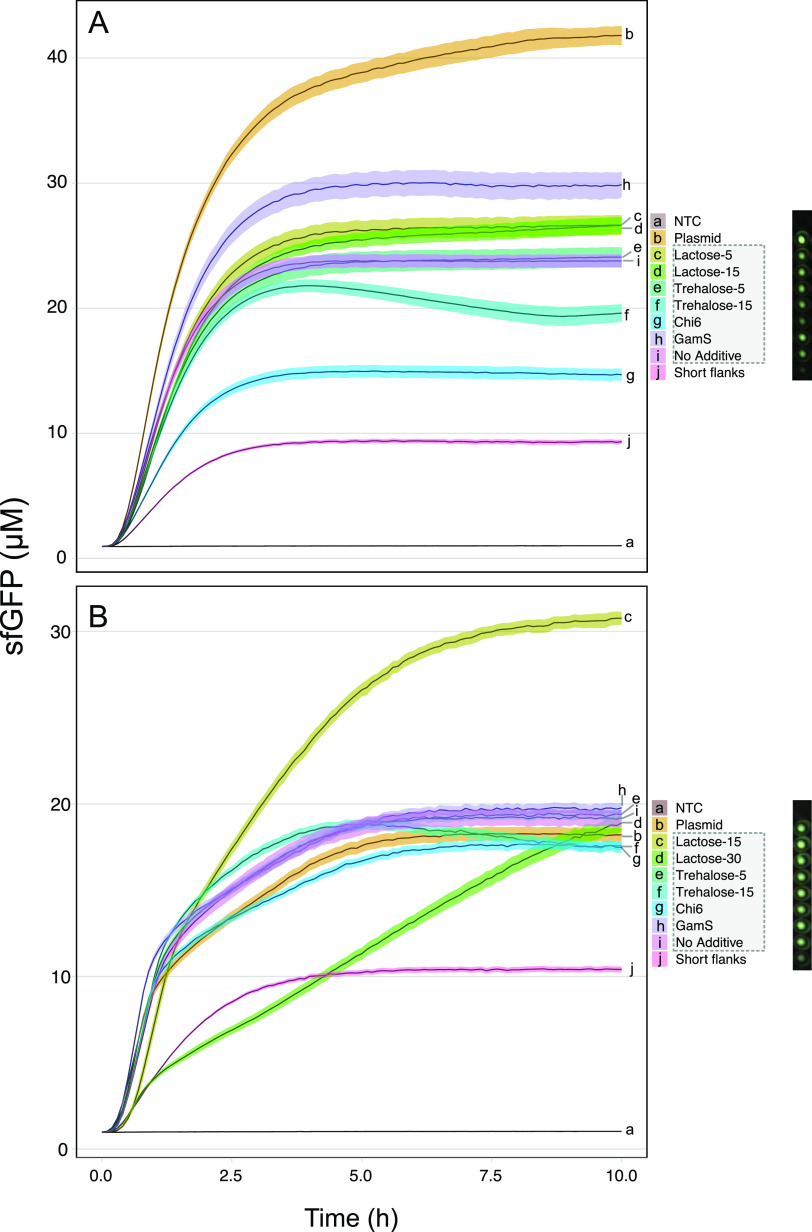
Enhancer effect of lactose on gene expression using linear DNA templates in cell-free reactions. CFPS based on (A) PEP or (B) MDX. Details of additives in each cell-free mixture are shown in Table S5. GamS and Chi6 were added at a final concentration of 2 μM. Except for NTC (no template control), all reactions contained 5 nM DNA (plasmid or linear). Linear DNA templates were amplified with extended 100 bp flanks to protect the template (highlighted with a gray dashed box). Unprotected linear DNA template was amplified with extended 3 bp flanks (denoted as “short flanks”). Black strips are representative images of the fluorescence signal on the plate captured using an imaging system (BioRad GelDoc-Go). Cell-free reactions were incubated at 29 °C for 10 h. All error bars represent standard error over three biological replicates based on three technical measurements.

Linear DNAs can also act as templates for RNA and protein synthesis using cell-free technology. These DNA templates are a popular alternative to plasmids since their preparation is fast and convenient.^47^ However, protein synthesis yields can be low due to the endogenous exonuclease activity present in the cell extracts and degradation of DNA templates. To address this problem, strategies such as the use of Chi sequences,^25^ GamS,^21,47,48^ and polymerase chain reaction (PCR) products with long flanks^3^ have been used to stabilize linear DNAs. To determine whether the addition of lactose to either of our two fresh formulations (Tables S1 and S2) can improve the protein yields in cell-free reactions based on linear DNA templates, we tested the two best lactose concentrations from our previous experiments with fresh samples (Figures 2D,I and S2I). In addition, linear DNA templates with T7 promoter and terminator were amplified with extended 100 bp flanks to protect the template. We observed that extended flanking sequences stabilized linear templates in both cell-free formulations (Figure 3A,B), with the exception of those that were supplemented with Chi6 sequences or trehalose at higher concentration (11.2 mM) in the PEP formulation (Table S2). In the latter case, there was a slight improvement in the protein yields from 23 to 26 μM sfGFP when lactose was added, and the best performance (30 μM) was seen in samples treated with GamS. Surprisingly, the profile changed when maltodextrin was used in the reactions (Figure 3B). The addition of 13.7 mM of lactose showed the greatest yield improvement from 19 to 31 μM of sfGFP, even better than those obtained after supplementation with GamS (20 μM) or using plasmids as DNA templates in cell-free reactions based on this formulation (Table S1). Indeed, a similar boost in yields was seen when lactose was added to a commercial version of the cell-free extract (Linear DNA Expression Kit, MyTxTL 508024) (Figure S5), where maltodextrin is also used as an energy source in the commercial kit.^19^ The addition of lactose provides a general boost for protein production from linear DNAs protected with long flanks, achieving high protein titers (>30 μM sfGFP) in low-cost reaction mixtures (£0.044 per 12 μL reaction) consuming maltodextrin as a cheap energy source.

### Low-Cost Production of High-Value Protein Reagents

**Figure 4:**
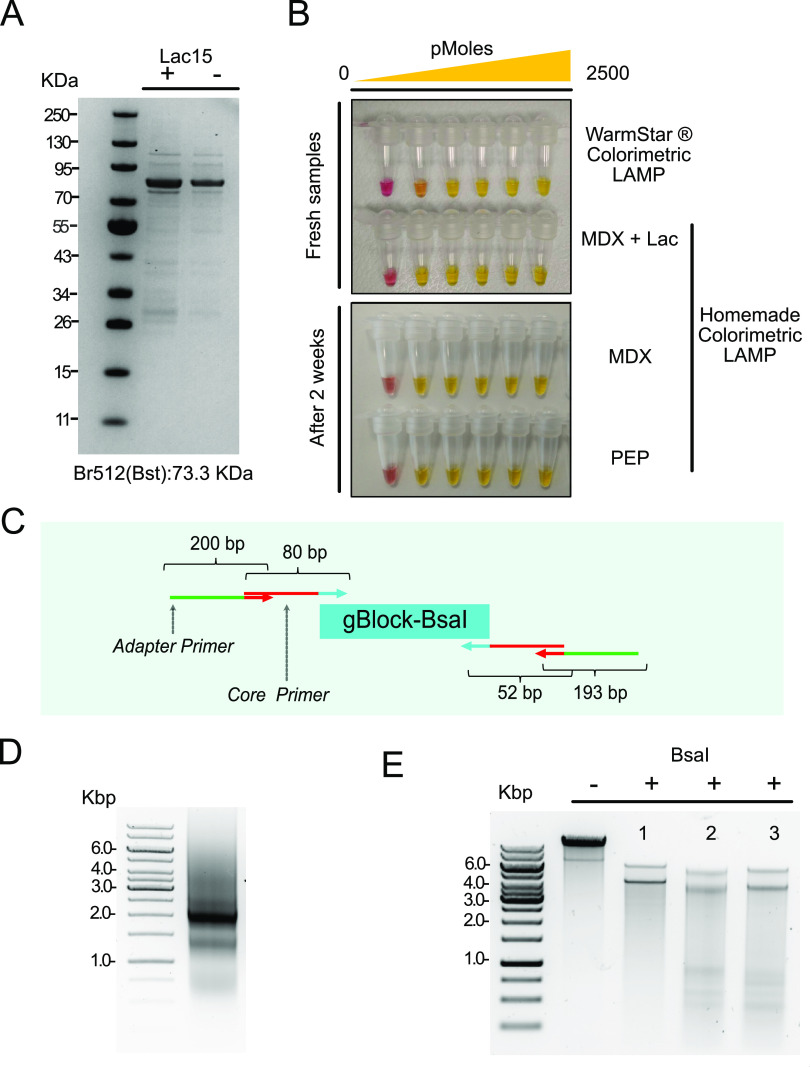
Production of molecular biology reagents. (A) Purified Br512 Bst DNA polymerase visualized in a polyacrylamide gel stained with coomassie blue. (B) Colorimetric LAMP assay using the Br512 Bst DNA polymerase produced *in vitro* in both fresh conditions (top panel) and using rehydrated samples (bottom panel) after a 2 week storage at room temperature. Cell-free reactions based on PEP and MDX were prepared using the low-cost drying system and protected with sucrose (120 and 15 mM, respectively). A synthetic dsDNA fragment from actin B gene (*Homo sapiens*) was used as a target in the following amounts: 0, 0.025, 0.25, 2.5, 250, and 2500 pmoles. Primers used in this assay are described in Table S11. Negative reactions were pink-colored, and positive reactions changed to yellow. (C, D) A PCR product encoding the *Bsa*I restriction endonuclease (2043 bp) was amplified using a single PCR with four oligonucleotides. An inner set of core primers provided a template for secondary amplification by longer oligonucleotides. The resulting product had extended terminal sequences that helped protect the coding region from exonuclease degradation. (E) Testing of *Bsa*I by restriction endonuclease digestion of luxpGEX plasmid. Digestion was performed using *Bsa*I produced by cell-free technology. Plasmid DNA samples were treated with (1) FastDigest Eco31I (Thermo Scientific, FD0293) (Isoschizomer: *Bsa*I), (2) *Bsa*I in cell extract, and (3) *Bsa*I in cell extract: 100% glycerol (1:1). Expected size of bands after digestion: 6440 and 4433 bp.

To test the utility of the expression systems described in this work, we expressed a modified version of *Bst* DNA polymerase^49^ (Br512; Figure 4A) using the improved formulation with MDX (Table S1; cell-free formulation based on maltodextrin supplemented with 13.7 mM lactose). *Bst* is an isothermal polymerase commonly used in loop-mediated isothermal amplification (LAMP) due to its high tolerance of clinical samples, and the enzyme is a useful component of rapid point-of-care diagnostic kits.^50,51^ The enzyme was produced by the transcription–translation of a plasmid template in 20 × 12 μL reactions, followed by pooling of the samples and affinity-column purification of the protein product. This simple procedure yielded 60.9 ± 0.5 μg of Bst DNA polymerase, and its activity was tested in a homemade colorimetric LAMP assay (Figure 4B, top panel), displaying equal effectiveness to the equivalent commercial assay. The procedure allowed the construction of a LAMP assay at a cost 20-fold cheaper than the commercial version. In addition, the approach reduced the need for specialized equipment, time, and effort generally required to produce this polymerase.^49,52^

We had demonstrated that it was possible to produce sfGFP from linear PCR-amplified DNAs without the requirement for expensive reagents to protect the templates against exonucleases (Figure 3). We decided to use our lactose-containing extracts (Table S1; 13.7 mM lactose) to express *Bsa*I, a type IIS restriction enzyme frequently used in Golden Gate cloning.^53^*Bsa*I (EcoR31I) is an example of a toxic protein that can only be expressed in special *E. coli* strains that are protected by the expression of the cognate methylase or similar.^54,55^ For this reason, it is difficult to obtain plasmid DNA templates encoding the gene.^56^ To side-step these problems, we used a chemically synthesized linear DNA template that was coamplified with 4-oligonucleotides (Figure 4C and Table S11). The flanking oligonucleotides included a set of two adapters for the particular target sequence and two longer sequences that could be reused (to avoid the costs of resynthesis). The final PCR product was 2043 bp size (Figure 4D), purified from 0.8% (w/v) agarose gel and used directly as a template for protein production, as described for Bst DNA polymerase. To verify its activity, a restriction analysis was performed using the enzyme product (Figure 4E), confirming the feasibility of producing active reagents like type IIS restriction enzymes through cell-free technology using synthetic dsDNA fragments that can be propagated by *in vitro* PCR.

To further challenge the system in the production of high-value reagents, cell-free reactions were prepared through the low-cost drying method described above (Tables S3 and S4) using PEP and MDX formulations and protected with 120 and 15 mM sucrose, respectively. After 2 weeks of storage at room temperature, 20 × 12 μL reactions were rehydrated with 5 nM of Br512 plasmid (for *Bst* expression) or 5 nM of linear DNA to produce *Bsa*I. Lactose was not included in the reactions since the enhancer effect of lactose was only preserved in fresh reactions (Figure S4). After an overnight incubation at 29 °C, this system yielded 37.0 ± 6.5 and 24.0 ± 0.7 μg of *Bst* DNA polymerase when PEP and MDX were used as energy sources, respectively. Using a homemade colorimetric LAMP assay (Figure 4B, bottom panel), the activity of Bst was tested showing a similar efficiency to when the polymerase was produced in fresh cell-free extracts. These results demonstrate the feasibility of producing protein reagents from dried cell-free reactions prepared by a low-cost drying system and using circular DNA as a template. However, when linear DNA was used as a template, the production of *Bsa*I was not possible under the tested conditions even when a nuclease inhibitor (2 μM GamS) was added in the reaction, probably due to the instability of the linear DNA in the new reaction environment created after sample rehydration.

### Deconstructing the Cell-Free Formulation

**Figure 5:**
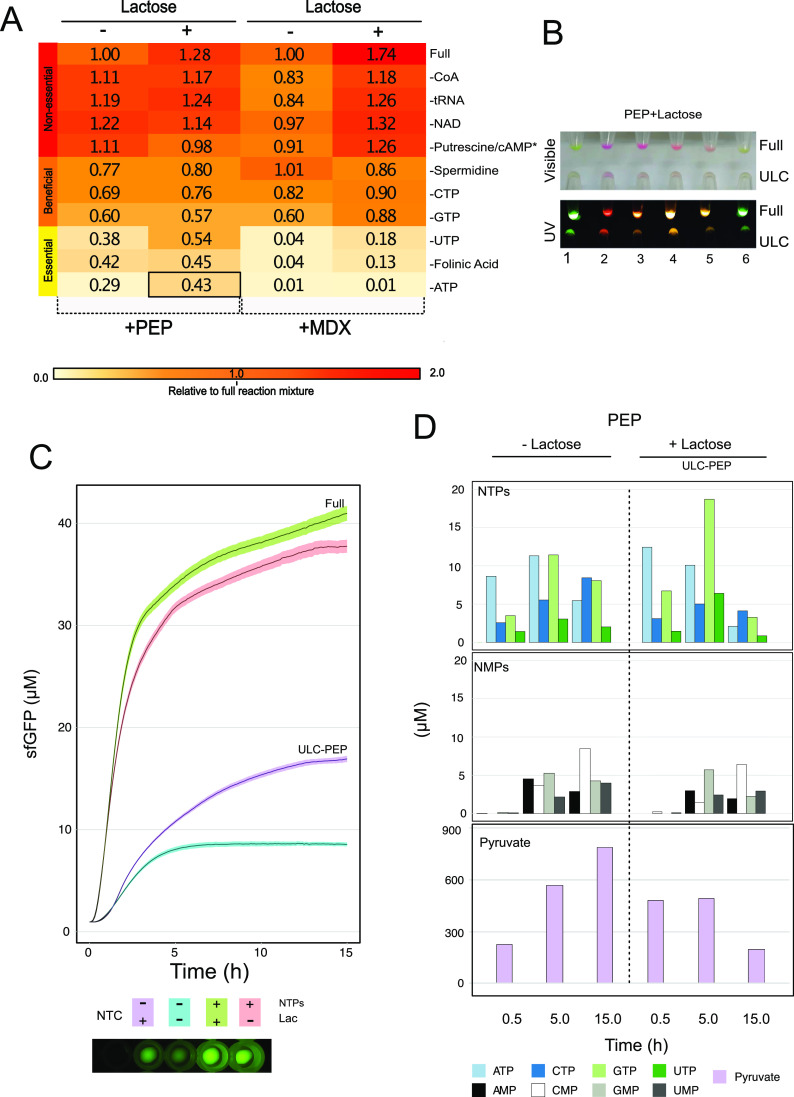
Ultra-low-cost (ULC) cell-free formulation based on PEP. (A) Relative levels of cell-free protein synthesis after the successive removal of reaction components according to Tables S7B and S8B. Every sequential row removes one more reagent in addition to those above it. Cell-free reactions were prepared with PEP or MDX as an energy source. The importance of additional components in the reaction buffers was tested by omission, starting with the most costly and less essential. The activities of the cell-free extracts were measured by sfGFP production and normalized relative to the respective full reaction (PEP/PEP complete reaction or MDX/MDX complete reaction). All measurements were based on three biological and three technical replicates. Relative level of protein synthesis for the ultra-low-cost (ULC) cell-free formulation is highlighted with a black square. (B) Synthesis of fluorescent proteins using the ULC cell-free formulation supplemented with 11.25 mM lactose (Table S13). Reporters: (1) psfGFP, (2) pJL1-eforRed, (3) pJL1-dTomato, (4) pFGC-T7-RibJ-mScarlet, (5) pFGC-T7-RibJ-RRvT, and (6) pFGC-T7-RibJ-mTFP1. (C) Quantification of sfGFP production in ULC-PEP formulation supplemented with 11.25 mM lactose. (D) Regeneration of NTPs, NMPs, and pyruvate during the cell-free reactions based on ULC-PEP formulation, measured by LC-MS at four time points. Samples were prepared as described in Table S13, replacing the indicated DNA with MQ water. Cell-free extracts were supplemented with 11.25 mM lactose, as indicated. Concentrations of nucleotides and pyruvate were measured by LC-MS.

To identify nonessential components in the cell-free mixture (Tables S7 and S8) and evaluate the enhancing effect of lactose in these reactions under fresh conditions, we successively removed each of the components present in the 25× nucleotide mix (Table S7A) and 10× energy buffer (Table S8A), which are used to prepare cell-free mixtures based on PEP and MDX energy sources. We started with the removal of the most expensive reagents, followed by elements thought to be essential.^41^ Our analysis allowed us to identify three groups of reagents that we categorized as nonessential, beneficial, or essential for the cell-free reactions (Figure 5A). In the first group, we observed a positive response when CoA, tRNA, NAD, putrescine, and cAMP (only included in the maltodextrin mix) were added in both formulations (Tables S7B and S8B). Reactions supplemented with MDX showed a drop in the range of 10–17% in relative yields of sfGFP protein, which recovered when 13.7 mM lactose was added (Figure 5A). For the second group of reaction components, spermidine, CTP, and GTP proved beneficial for both systems (Figure 5A). However, unlike the first group, yields did not recover on the addition of lactose. The removal of UTP or folinic acid from cell-free reactions based on maltodextrin resulted in the plunge of protein synthesis yields to 4% and total loss in the absence of ATP. Surprisingly, sfGFP protein yields remained at 50% when lactose (11.2 mM) was added to the PEP-based reactions (Figure 5A,B). Further, we did not observe a full loss of protein synthesis after completely removing all of these components, and the addition of lactose improved the yield from 29 to 43% (Figure 5A,C). A possible explanation is that the conversion from PEP to pyruvate is coupled to nucleotide regeneration and mRNA translation.^43,57^ To investigate this, the concentrations of NTPs, NMPs, and pyruvate were measured by liquid chromatography–mass spectrometry (LC–MS) (Table S12 and Figure S6) in those samples devoid of external sources of nucleotides but fed with either PEP or MDX. Our results demonstrated that the ability to regenerate nucleotides (NMPs, NTPs) and pyruvate in cell-free reactions is related to the addition of an energy source since when PEP is removed from the mixture (Figure S7A), the concentration of these metabolites was depleted. In contrast, when PEP was added into the mixture, our results indicated endogenous biosynthesis of NTPs (Figure 5D, upper panel) during the CF reaction due to pyruvate formation (Figure 5D, lower panel), while high levels of GTP (18.69 μM) and ATP (12.43 μM) were seen. This is consistent with the requirement for these nucleotides in mRNA translation, where two GTPs are required for each cycle of aminoacyl-tRNA delivery and ribosome translocation and one ATP is required for peptide bond formation.^13,57^ Interestingly, the addition of MDX (supplemented or not with lactose) in CF reactions also sustains NTP and pyruvate biosynthesis (Figure 7SB), recording higher levels of GTP (30.6 μM) and ATP (44.9 μM) than in the PEP formulation when lactose and MDX are included in the reaction. This is consistent with our previous results (Figure 3B), where protein production is enhanced in cell-free reactions based on MDX and supplemented with lactose as additive. However, it does not explain the observed inability of maltodextrin to sustain protein production when exogenous nucleotides are omitted from the reactions (Figure 5A). We speculate that the glycolytic pathway and nucleotide pool were affected by lack of crowding agents and other additives missing from the maltodextrin system (Table S8), which may delay the synthesis of NTPs, since in the PEP system the maximum GTP concentration was observed after 5 h of incubation, while in MDX formulation, the maximum was registered after 15 h (Figures 5D and S7B). In addition, the fast consumption of pyruvate and the absence of some NMPs such as GMP in the CF reactions supplemented with MDX seem to compromise the activity of PANOx system, which is coupled to the maltodextrin system.^15,58^ In fact, an increment in the AMP concentration and a reduction in the ATP levels were measured, suggesting a reconversion from the triphosphate to monophosphate form (Figure S7B), which agrees with previous studies where cell-free systems based on glucose as an energy source and fed with NMPs did not result in protein synthesis but showed conversion of ATP to AMP.^59^ Accordingly, protein synthesis was only possible in the PEP system, where, with the enhancing effect of lactose, a production of 16.92 μM sfGFP was observed.

Overall, our combined findings suggest that it is possible to reduce the cost of cell-free formulations (Table S14), especially for use in an educational context or other low-resource settings, since the activities of these simpler extracts are high enough for detection in the classroom (Figure 5B). So far, we have achieved an over 400-fold reduction in reaction costs compared to commercial versions and almost a 3-fold reduction compared to the cost of DIY reactions (Figure S8). This is due to the use of cheaper sources of PEP (22.2 times less expensive than other suppliers) (Figure S8A,B,D,E) and savings due to the removal of the nucleotide mix, which represents 45.84% (Figure S8A,F) in the total cost of a reaction. Our study demonstrated a cell-free formulation completely free of external NTPs, which is even cheaper than the maltodextrin system using PEP from a different supplier (Figure S8D,E). This extends previous attempts to build economical and open methods for programmable biosynthesis.^11,18,60−63^

## Conclusions

Cell-free technologies offer many potential uses for education, research, and point-of-care and field applications in LMICs, and numerous efforts have been made to improve access to these tools by removing cost barriers in manufacturing.^2,11,16,18,64^ Despite much progress, improvements are still required. Here, we describe several advances. First, we developed a cost-effective platform (USD $177) based on silica beads and a conventional low-vacuum source to dry cell-free reactions without the use of expensive equipment (>USD $10 000). Using this platform, we achieved up to 19 μM sfGFP protein production after 2 weeks of storage at room temperature. While the use of silica for drying cells has been previously reported,^65^ here we report the first use for preserving fully assembled cell-free reactions ready for cold-chain-free distribution and use. Second, we describe the enhancing effect of lactose in cell-free formulations, obtaining a substantial improvement in reactions that use maltodextrin as an energy source. This improvement allowed us to use lactose as an additive for expressing proteins using linear DNA templates without the addition of costly stabilizers such as GamS. Finally, we demonstrated that protein synthesis is sustainable in cell extracts without adding an external source of NTPs. We believe that this is the forerunner of future work to more deliberately exploit regeneration systems in cell-free reactions to further lower costs and pave the way for a wider use of these systems in low-resource contexts.

## Methods

### Molecular Biology

Unless otherwise stated, all PCR reactions were performed using a Q5 High-Fidelity 2× Master Mix (New England Biolabs, M0492S) according to the manufacturer’s instructions. For a single PCR reaction using four primers simultaneously, the reaction mix was composed of 0.5 μM of each adapter primer, 0.025 μM each core primer (20× less concentrated than adapter primers), 40–60 ng of DNA template, and 1× Q5 High-Fidelity Master Mix. PCR conditions are described in Table S10. Cell-free backbone plasmid was synthesized by IDT (Integrated DNA Technologies) using kanamycin as a resistance marker. New plasmids were constructed using conventional PCR products or double-stranded DNA fragments (Genewiz, U.K.) along with the destination pFGC-T7-RJBB plasmid (vector backbone with the T7 promoter-RiboJ- *Bsa*I-LacZα-*Bsa*I-T7 terminator configuration) in a single Golden Gate cloning reaction. All of the molecular cloning steps and plasmid propagation were performed in *E. coli* Top10 (Invitrogen, C404010). DNA plasmid for cell-free reactions was obtained by midi-prepping (Sigma-Aldrich, NA0200-1KT) an overnight culture of 50 mL LB with the appropriate strain and antibiotic according to the fabricant’s instructions. Plasmids and primers are listed in Tables S9 and S11 respectively. Coding sequences cloned into the pFGC-T7-RJBB plasmid are described in Table S12. Plasmids are available at Addgene (173224-27).

### Extract Preparation

To prepare crude cell extracts, 5 μL of BL21 Star glycerol stock (Invitrogen, C601003) was inoculated into 5 mL of 2xYT medium. The preculture was grown for 8 h at 37 °C with 200 rpm shaking. Afterward, 50 mL of 2xYT medium was inoculated with 30 μL preculture in a 250 mL flask and grown at 37 °C with vigorous agitation (200 rpm). The next day, the stationary phase preculture was used to inoculate 400 mL of 2xYT media supplemented with 18 g/L d-g glucose in a 2.5 L baffled Tunair flask (Sigma-Aldrich, Z710822), giving an initial optical density (OD600) of 0.05. Cultures were grown at 37 °C with shaking (200 rpm) until OD600 reached 0.5 (approximately 2.5 h), and then the cells were induced with 400 μL of 1 M IPTG. Cells were harvested in the exponential phase at an optical density (OD600) of 2.0 by centrifugation at 5000*g* and 4 °C for 12 min. Pellets were washed three times with S30A buffer (50 mM Tris base, 14 mM magnesium glutamate, 60 mM potassium glutamate, 2 mM dithiothreitol (DTT), pH 7.7 adjusted with 1:1 acetic acid). Afterward, the pellet was weighed and resuspended in 0.9 mL of S30B buffer (5 mM Tris base, 14 mM magnesium glutamate, 60 mM potassium glutamate, 1 mM dithiothreitol, pH 8.2 adjusted with 1:1 acetic acid) per gram of pellet. Cell suspension was distributed in 1 mL aliquots in 1.5 microcentrifuge tubes and then lysed by sonication on a QSonica Q125 sonicator with a 3.175 mm diameter probe, as previously described by Silverman et al.^66^ at a frequency of 20 kHz and 50% amplitude by 10 s ON/OFF pulses for a total of 60 s (delivering ∼350 J). The lysate was centrifuged for 10 min at 4 °C and 10 000*g*. To clarify the cell extracts, the supernatants were centrifuged a second time for 15 min at 4 °C and 12 000*g*. Finally, the crude extracts were pooled, supplemented with 1 mM DTT, aliquoted, and snap-frozen in liquid nitrogen.

### Cell-Free Reactions

A typical cell-free reaction based on PEP as energy source was composed of 175 mM potassium glutamate, 10 mM ammonium glutamate, 2.7 mM potassium oxalate, 1 mM putrescine, 1.5 mM spermidine, 0.33 mM NAD, 1.2 mM ATP, 0.86 mM CTP, GTP and UTP, 0.27 mM CoA, 0.172 mg/mL of MRE600 *E. coli* tRNA, 0.07 mM folinic acid, 33 mM PEP, 2 mM of each of the 19 amino acids (glutamate was omitted since it is already present in the reaction mixture as potassium glutamate), 10 mM magnesium glutamate, 2% (w/v) PEG-8000, 5 nM DNA (plasmid or linear), and 33.33% (v/v) of crude extract by volume. For reactions using maltodextrin (MDX) as energy source, the cell-free mixture is composed of 50 mM HEPES pH 8, 1.5 mM ATP and GTP, 1.4 mM CTP and UTP, 0.2 mg/mL tRNA, 0.26 mM CoA, 0.33 mM NAD, 0.76 mM cAMP, 0.01 mM folinic acid, 0.11 mg/mL spermidine, 2% (w/v) PEG-8000, 3.4 mM of each of the 19 amino acids, 12 mg/mL maltodextrin, 0.60 mg/mL sodium hexametaphosphate, 2.6 mM magnesium glutamate, 56 mM potassium glutamate, 5 nM DNA (plasmid or linear DNA), and 33.33% (v/v) of crude extract by volume. An ultra-low-cost cell-free reaction contains 175 mM potassium glutamate, 10 mM ammonium glutamate, 2.7 mM potassium oxalate, 33 mM PEP, 2 mM of each of the 19 amino acids, 10 mM magnesium glutamate, 2% (w/v) PEG-8000, 5 nM DNA (plasmid or linear), 11.25 mM lactose as enhancer, and 33.33% (v/v) of crude extract by volume. Detailed protocols for preparing all cell-free stock solutions used in this study are available at protocols.io/researchers/fernando-guzman-chavez.

### Lyophilization and Silica Drying of Cell-Free Reactions

Unless otherwise specified, all of the lyophilization mixes contain the composition described above for cell-free reactions using either PEP or MDX as an energy source, excluding the DNA and adding the different cryoprotectants at the tested concentrations (0, 5, 15, 30, 60, and 120 mM) in the lyophilization mix (refer to Tables S1–S6 for more details in the cell-free reaction compositions). This mix was distributed in 96-well PCR plates (4titude, 4ti-1000/R) in 9 μL aliquots for mixes containing PEP and 11 μL aliquots for those with MDX. Using different wells in the same plate, 20 nM psfGFP plasmid^17^ was distributed in 9 μL volumes along the plate. Afterward, the 96-well plate was sealed with adhesive aluminum foil seals (4titude, 4ti-0550) and punctured with a 16G needle to create one hole. For lyophilization, the samples were frozen at −80 °C for 30 min and then placed in a FreeZone Triad Benchtop Freeze Dry System (Labconco), previously cooled reaching a condenser temperature of −80 °C. Then, the samples were freeze-dried following a three-step program: 12 h at −45 °C, 10 h at −5 °C, and 4 h at 20 °C with a constant pressure of 0.04 mbar throughout the process. The temperatures indicated in the three-step program correspond to shelf temperatures inside of the chamber.

For low-cost drying, the samples were transferred to a low-cost drying device (Figure 1A), which consisted of a Nalgene Desiccator (ThermoFisher, 5311-0250PK) with 500 g of silica gel (Fisher Scientific, S/0761/53) connected to the laboratory vacuum system. The samples were left to dry overnight at room temperature under vacuum. The next day, the plate was sealed with adhesive aluminum foil seals (4titude, 4ti-0550) and punctured with a 16G needle to create one hole.

In both cases and unless rehydrated immediately, freeze-dried reactions were packaged as previously described by Jung et al. 2020^23^ with the following modifications: the dried samples were placed into a vacuum sealer bag (12 cm × 16 cm Vacuum Food Sealer Embossed Bags, Amazon, Amazon Standard Identification Number (ASIN) B015A7LH9A) with two desiccant packs (2 g Small Silica Gel Sachets, Amazon, ASIN: B07PRGC434), two oxygen absorbers (Fresherpack 20cc Oxygen Absorbers, Amazon, ASIN: B00U2O3VAK), purged with argon using an argon canister (Preservintage Wine Preserver, Amazon, ASIN: B07MQFTKPN), and impulse-heat-sealed (Audew Food Vacuum Sealer, Amazon, ASIN: B07QC2BTJ9). Afterward, the samples were placed in a second light-protective bag (Open Top Mylar Foil Aluminium Bag, Amazon, ASIN: B01MY95ICS) and impulse-heat-sealed.

### Fluorescence Quantification

Fresh cell-free reactions were prepared as described in Tables S1 and S2. Dried samples of DNA plasmid were rehydrated with 36 μL of PCR-grade sterile water (MQ) to produce a concentration of 5 nM. Cell-free pellets were reconstituted with 12 μL of the plasmid solution and incubated at room temperature for 1 min. According to the experiment, 10 μL of either fresh or rehydrated samples was loaded into V-bottom 96-well plates (Corning, CLS3957). Reactions were incubated in a CLARIOStar plate reader (BMG Labtech, Germany) at 29 °C, and fluorescence measurements (emission/excitation: 470/515 nm; gain = 500) were recorded every 6 min for 18 h. To quantify fluorescent protein concentrations, recombinant eGFP standard (Cell Biolabs, STA-201) was used to create a calibration curve.

### Cell-Free Reactions Using Linear DNA

Cell-free reactions were set up as described above. When required, GamS and Chi6 (exonuclease inhibitors) were added to a final concentration of 2 μM^48^ while the corresponding volume of water in the cell-free reaction mix was adjusted (Tables S3 and S4). Linear templates were prepared by PCR amplification and purified from 0.8% (w/v) agarose gel using Monarch DNA Gel Extraction Kit (New England Biolabs, T1020S), according to the manufacturer’s protocol. When psfGFP plasmid was used as a template, purified PCR products were treated with FastDigest *Dpn*I restriction enzyme (Thermo Scientific, FD1704) for 30 min at 37 °C to cut methylated DNA to eliminate the DNA template. Afterward, the samples were purified using Monarch PCR & DNA Gel Cleanup Kit (New England Biolabs, T1030S) and quantified using a nanodrop spectrophotometer (Thermo Scientific, NanoDrop One).

### Removing Reagents in Cell-Free Reactions

To study the effect of different constituents in the cell-free composition, 25× nucleotide mix and 10× energy were modified according to Tables S7 and S8. In all cases, omitted reagents were replaced with the equivalent volume of MQ water and adjusted to pH 7.5. To perform either a cell-free reaction based on PEP or MDX, the modified versions were used to prepare a 4× wizard mix or 2.5× reaction buffer instead of the complete version. Reactions were assembled according to Tables S1 and S2. All of the reactions were incubated at 29 °C, and measurements were recorded every 6 min for 18 h.

### Cell-Free Production of *Bsa*I Enzyme

To produce *Bsa*I enzyme, 20 cell-free reactions (12 μL per reaction) based on maltodextrin and supplemented with lactose (13.7 mM; Tables S1 and S5) were performed using DNA templates; the PCR product was amplified from a double-stranded DNA fragment (gBlock; Table S12), purified from 0.8% (w/v) agarose gel, and incubated overnight at 29 °C. To generate the PCR fragment, a 4 oligos PCR approach was performed as above described. The next day, the samples were pooled, diluted 1:1 with 100% glycerol, and stored at −20 °C. To test the activity, 400 ng pGEX-ilux plasmid was digested in a homemade CutSmart Buffer (50 mM potassium acetate, 20 mM Tris-acetate, 10 mM magnesium acetate, 100 μg/mL bovine serum albumin (BSA), pH 8) at 37 °C for 1 h. The digested plasmid was cleaned using a Monarch PCR & DNA Cleanup kit (NEB, T1030S) following the manufacturer’s instructions and visualized in a 0.8% (w/v) agarose gel. The undigested plasmid was used as a control.

### LAMP Colorimetric Assay

To perform LAMP assays, Br512 (a modified version of B*st* DNA polymerase) was produced using cell-free technology. In short, 20 reactions (12 μL per reaction) were performed using the plasmid pKAR2-Br512^49^ (Addgene: 161875) as DNA template either in a fresh cell-free mixture based on maltodextrin and supplemented with lactose (13.7 mM; Tables S1 and S5) or in rehydrated samples after 2 weeks of storage at room temperature. Dry samples were prepared through the low-cost drying system as described above, using PEP and MDX as energy sources and protected with sucrose (120 and 15 mM, respectively). Reactions were incubated overnight at 29 °C. The next day, the 20 reactions were pooled and purified using a Ni-NTA Spin Column (Qiagen, 31314) according to the manufacturer’s instructions. The sample was eluted in 300 μL of elution buffer (50 mM NaH_2_PO_4_, 300 mM NaCl, 500 mM imidazole, pH 8) and diluted by adding 200 μL of buffer A (50 mM NaH_2_PO_4_, 50 mM NaCl). Afterward, the sample was concentrated and the buffer was exchanged using a 3K Amicon filter (Merck, UFC500324) with storage buffer (50 mM NaH_2_PO_4_, 50 mM NaCl, 2 mM DTT, 0.2 mM EDTA, 0.2% Triton X-100, pH 8). The purified protein from 20 cell-free reactions (12 μL per reaction) was quantified using Pierce 660 nm Protein assay (Thermo Scientific, 22660), visualized in a coomassie blue polyacrylamide gel, and then diluted 1:1 with 100% glycerol and stored at −20 °C. LAMP colorimetric reactions were prepared in 25 μL volume containing 1× colorimetric buffer^67^ (10 mM (NH_4_)_2_SO_4_, 50 mM KCl, 2 mM MgSO_4_, 0.1% Tween 20, 0.1 mM Cresol Red, pH 8.8), DNA template (actin B; Table S12), 6 mM MgSO_4_, 1.4 mM dNTPs, Br512 purified protein (10 pmoles), 1.6 μM each FIP and BIP primers, 0.2 μM each F3 and B3 primers, and 0.4 μM each loop primers (Table S11). Reactions were incubated at 65 °C for 30 min in a thermocycler (Applied Biosystems, ProFlex PCR system). WarmStart Colorimetric LAMP 2× Master Mix (New England Biolabs, M1800S) was used as a control to evaluate the efficiency in the colorimetric LAMP assay.

### Quantification of Nucleotides and Pyruvate by LC-MS

To quantify nucleotides and pyruvate, an equivalent volume of 20 cell-free reactions (12 μL per reaction) was prepared, as described in Table S13, replacing the indicated volume of DNA with MQ water. CF reactions were supplemented with 11.2 mM lactose as indicated. Samples were incubated for 0, 0.5, 5, and 15 h at 29 °C and then were analyzed by LC-MS using the protocol described in Vilkhovoy et al.^68^ Briefly, the samples were first deproteinized by adding an equal volume of ice-cold 100% ethanol. This mixture was centrifuged at 12 000*g* for 15 min at 4 °C, and the supernatant fraction, which contained the metabolites, was collected and diluted 5-fold in ultrapure water to a volume of 50 μL. To tag the samples with aniline, 5 μL each of EDC (200 mg/mL) and 12C aniline were added to the mixture (13C in the case of internal standards), and the reaction was mixed at room temperature for 2 h by gentle shaking. The tagging reaction was quenched by adding 1.5 μL of triethylamine and centrifuging the mixture at 13 500*g* for 3 min. Twenty-five microliters each of the tagged internal standard and tagged samples were mixed and then analyzed by the LC-MS system. LC separation was performed on an Acquity BEH C18 Column (1.7 μm, 2.1 mm × 150 mm) at a flow rate of 0.3 mL/min and an injection volume of 5 μL. The elution started from 95% mobile phase A (5 mM tributylamine (TBA) aqueous solution, adjusted to pH 4.75 with acetic acid) and 5% mobile phase B (5 mM TBA in acetonitrile), increased to 70% B in 10 min, further increased to 100% B in 2 min, held at 100% B for 2 min, returned to initial conditions over 0.1 min, and held for 4 min to re-equilibrate the column. The mass spectrometer was set to a negative ion mode with a probe temperature of 520 °C, a negative capillary voltage of −0.8 kV, a positive capillary voltage of 0.8 kV, and an acquisition range of *m*/*z* 130–900.

## Supporting Information Available

Lyoprotectant effects of five sugars individually added to the two cell-free formulations (Figure S1); lyoprotectant effects of five sugars individually added in higher concentrations (Figure S2); sharing lyophilized and dried cell-free reactions around the globe (Figure S3); effect of lactose in cell-free reactions before and after being added during the lyophilization/drying process (Figure S4); enhancing effect of lactose over sfGFP production in fresh cell-free reactions in three different formulations (PEP, MDX, or commercial version) (Figure S5); chromatograms of NTPs and pyruvate detection by LC-MS at four time points (Figure S6); regeneration of NMPs, NTPs, and pyruvate during the cell-free reactions (Figure S7); cost comparison between five different cell-free formulations (Figure S8);
composition of fresh MDX-based energy mix (Table S1); composition of fresh PEP-based energy mix (Table S2); composition of MDX-based energy mix for lyophilization (Table S3); composition of PEP-based energy mix for lyophilization (Table S4); sugar concentrations used in fresh and lyophilized extracts (Table S5); source of sugars used in this study (Table S6); composition of 25× nucleotide mix variants (Table S7); composition of 10× nucleotide mix variants (Table S8); plasmids used in this study (Table S9); PCR conditions (Table S10); primers used in this study (Table S11); sequences used in this study (Table S12); and ultra-low-cost (ULC) formulation for fresh PEP-based cell-free reactions (Table S13) (PDF)
Cost breakdown (Table S14) (XLSX)

## References

[ref1] Nirenberg M. W.; Matthaei J. H. The dependence of cell-free protein synthesis in *E. coli* upon naturally occurring or synthetic polyribonucleotides. Proc. Natl. Acad. Sci. U.S.A. 1961, 47, 1588–1602. 10.1073/pnas.47.10.1588. 14479932 PMC223178

[ref2] Gregorio N. E.; Levine M. Z.; Oza J. P. A User’s Guide to Cell-Free Protein Synthesis. Methods Protoc. 2019, 2, 24 10.3390/mps2010024. PMC6481089 31164605

[ref3] Ouyang X.; Zhou X.; Lai S. N.; Liu Q.; Zheng B. Immobilization of Proteins of Cell Extract to Hydrogel Networks Enhances the Longevity of Cell-Free Protein Synthesis and Supports Gene Networks. ACS Synth. Biol. 2021, 10, 749–755. 10.1021/acssynbio.0c00541. 33784075

[ref4] Matsuda T.; Ito T.; Takemoto C.; Katsura K.; Ikeda M.; Wakiyama M.; Kukimoto-Niino M.; Yokoyama S.; Kurosawa Y.; Shirouzu M. Cell-free synthesis of functional antibody fragments to provide a structural basis for antibody-antigen interaction. PLoS One 2018, 13, e0193158 10.1371/journal.pone.0193158. 29462206 PMC5819829

[ref5] Tinafar A.; Jaenes K.; Pardee K. Synthetic Biology Goes Cell-Free. BMC Biol. 2019, 17, 64 10.1186/s12915-019-0685-x. 31395057 PMC6688370

[ref6] Lavickova B.; Maerkl S. J. A Simple, Robust, and Low-Cost Method To Produce the PURE Cell-Free System. ACS Synth. Biol. 2019, 8, 455–462. 10.1021/acssynbio.8b00427. 30632751

[ref7] Shimizu Y.; Inoue A.; Tomari Y.; Suzuki T.; Yokogawa T.; Nishikawa K.; Ueda T. Cell-free translation reconstituted with purified components. Nat. Biotechnol. 2001, 19, 751–755. 10.1038/90802. 11479568

[ref8] Yang W. C.; Patel K. G.; Wong H. E.; Swartz J. R. Simplifying and streamlining Escherichia coli-based cell-free protein synthesis. Biotechnol. Prog. 2012, 28, 413–420. 10.1002/btpr.1509. 22275217

[ref9] Kim T. W.; Keum J. W.; Oh I. S.; Choi C. Y.; Kim H. C.; Kim D. M. An economical and highly productive cell-free protein synthesis system utilizing fructose-1,6-bisphosphate as an energy source. J. Biotechnol. 2007, 130, 389–393. 10.1016/j.jbiotec.2007.05.002. 17566582

[ref10] Calhoun K. A.; Swartz J. R. Energy systems for ATP regeneration in cell-free protein synthesis reactions. In In Vitro Transcription and Translation Protocols; Methods in Molecular Biology; Humana Press, 2007; Vol. 375, pp 3–17. 10.1007/978-1-59745-388-2_1 17634594

[ref11] Calhoun K. A.; Swartz J. R. An economical method for cell-free protein synthesis using glucose and nucleoside monophosphates. Biotechnol. Prog. 2005, 21, 1146–1153. 10.1021/bp050052y. 16080695

[ref12] Kim H. C.; Kim D. M. Methods for energizing cell-free protein synthesis. J. Biosci. Bioeng. 2009, 108, 1–4. 10.1016/j.jbiosc.2009.02.007. 19577183

[ref13] Whittaker J. W. Cell-free protein synthesis: the state of the art. Biotechnol. Lett. 2013, 35, 143–152. 10.1007/s10529-012-1075-4. 23086573 PMC3553302

[ref14] Kim T. W.; Oh I. S.; Keum J. W.; Kwon Y. C.; Byun J. Y.; Lee K. H.; Choi C. Y.; Kim D. M. Prolonged cell-free protein synthesis using dual energy sources: Combined use of creatine phosphate and glucose for the efficient supply of ATP and retarded accumulation of phosphate. Biotechnol. Bioeng. 2007, 97, 1510–1515. 10.1002/bit.21337. 17238210

[ref15] Wang Y.; Zhang Y. H. Cell-free protein synthesis energized by slowly-metabolized maltodextrin. BMC Biotechnol. 2009, 9, 58 10.1186/1472-6750-9-58. 19558718 PMC2716334

[ref16] Caschera F.; Noireaux V. A cost-effective polyphosphate-based metabolism fuels an all E. coli cell-free expression system. Metab. Eng. 2015, 27, 29–37. 10.1016/j.ymben.2014.10.007. 25446973

[ref17] Arce A.; Guzman Chavez F.; Gandini C.; Puig J.; Matute T.; Haseloff J.; Dalchau N.; Molloy J.; Pardee K.; Federici F. Decentralizing Cell-Free RNA Sensing With the Use of Low-Cost Cell Extracts. Front. Bioeng. Biotechnol. 2021, 9, 727584 10.3389/fbioe.2021.727584. 34497801 PMC8419261

[ref18] Rolf J.; Rosenthal K.; Lütz S. Application of Cell-Free Protein Synthesis for Faster Biocatalyst Development. Catalysts 2019, 9, 190 10.3390/catal9020190.

[ref19] Garenne D.; Beisel C. L.; Noireaux V. Characterization of the all-E. coli transcription-translation system myTXTL by mass spectrometry. Rapid Commun. Mass Spectrom. 2019, 33, 1036–1048. 10.1002/rcm.8438. 30900355

[ref20] Pardee K. Perspective: Solidifying the impact of cell-free synthetic biology through lyophilization. Biochem. Eng. J. 2018, 138, 91–97. 10.1016/j.bej.2018.07.008. 30740032 PMC6358126

[ref21] Didovyk A.; Tonooka T.; Tsimring L.; Hasty J. Rapid and Scalable Preparation of Bacterial Lysates for Cell-Free Gene Expression. ACS Synth. Biol. 2017, 6, 2198–2208. 10.1021/acssynbio.7b00253. 28795570 PMC6038143

[ref22] Pardee K.; Green A. A.; Takahashi M. K.; Braff D.; Lambert G.; Lee J. W.; Ferrante T.; Ma D.; Donghia N.; Fan M.; Daringer N. M.; Bosch I.; Dudley D. M.; O’Connor D. H.; Gehrke L.; Collins J. J. Rapid, Low-Cost Detection of Zika Virus Using Programmable Biomolecular Components. Cell 2016, 165, 1255–1266. 10.1016/j.cell.2016.04.059. 27160350

[ref23] Jung J. K.; Alam K. K.; Verosloff M. S.; Capdevila D. A.; Desmau M.; Clauer P. R.; Lee J. W.; Nguyen P. Q.; Pasten P. A.; Matiasek S. J.; Gaillard J. F.; Giedroc D. P.; Collins J. J.; Lucks J. B. Cell-free biosensors for rapid detection of water contaminants. Nat. Biotechnol. 2020, 38, 1451–1459. 10.1038/s41587-020-0571-7. 32632301 PMC7718425

[ref24] Sitaraman K.; Esposito D.; Klarmann G.; Le Grice S. F.; Hartley J. L.; Chatterjee D. K. A novel cell-free protein synthesis system. J. Biotechnol. 2004, 110, 257–263. 10.1016/j.jbiotec.2004.02.014. 15163516

[ref25] Marshall R.; Maxwell C. S.; Collins S. P.; Beisel C. L.; Noireaux V. Short DNA containing chi sites enhances DNA stability and gene expression in E. coli cell-free transcription-translation systems. Biotechnol. Bioeng. 2017, 114, 2137–2141. 10.1002/bit.26333. 28475211 PMC5522353

[ref26] Katzen F.; Chang G.; Kudlicki W. The past, present and future of cell-free protein synthesis. Trends Biotechnol. 2005, 23, 150–156. 10.1016/j.tibtech.2005.01.003. 15734558

[ref27] Corveleyn S.; Remon J. P. Maltodextrins as lyoprotectants in the lyophilization of a model protein, LDH. Pharm. Res. 1996, 13, 146–150. 10.1023/A:1016006106821. 8668665

[ref28] García-Coronado P.; Flores-Ramirez A.; Grajales-Lagunes A.; Godinez-Hernandez C.; Abud-Archila M.; Gonzalez-Garcia R.; Ruiz-Cabrera M. A. The Influence of Maltodextrin on the Thermal Transitions and State Diagrams of Fruit Juice Model Systems. Polymers 2020, 12, 2077–13. 10.3390/polym12092077. PMC7570093 32932726

[ref29] Flores-Ramírez A. J.; García-Coronado P.; Grajales-Lagunes A.; García R. G.; Abud Archila M.; Ruiz Cabrera M. A. Freeze-Concentrated Phase and State Transition Temperatures of Mixtures of Low and High Molecular Weight Cryoprotectants. Adv. Polym. Technol. 2019, 2019, 1–11. 10.1155/2019/5341242.

[ref30] Arakawa T.; Timasheff S. N. Stabilization of protein structure by sugars. Biochemistry 1982, 21, 6536–6544. 10.1021/bi00268a033. 7150574

[ref31] Carpenter J. F.; Prestrelski S. J.; Arakawa T. Separation of freezing- and drying-induced denaturation of lyophilized proteins using stress-specific stabilization. I. Enzyme activity and calorimetric studies. Arch. Biochem. Biophys. 1993, 303, 456–464. 10.1006/abbi.1993.1309. 8512328

[ref32] Starciuc T.; Malfait B.; Danede F.; Paccou L.; Guinet Y.; Correia N. T.; Hedoux A. Trehalose or Sucrose: Which of the Two Should be Used for Stabilizing Proteins in the Solid State? A Dilemma Investigated by In Situ Micro-Raman and Dielectric Relaxation Spectroscopies During and After Freeze-Drying. J. Pharm. Sci. 2020, 109, 496–504. 10.1016/j.xphs.2019.10.055. 31678247

[ref33] Kaushik J. K.; Bhat R. Why is trehalose an exceptional protein stabilizer? An analysis of the thermal stability of proteins in the presence of the compatible osmolyte trehalose. J. Biol. Chem. 2003, 278, 26458–26465. 10.1074/jbc.M300815200. 12702728

[ref34] Gregorio N. E.; Kao W. Y.; Williams L. C.; Hight C. M.; Patel P.; Watts K. R.; Oza J. P. Unlocking Applications of Cell-Free Biotechnology through Enhanced Shelf Life and Productivity of E. coli Extracts. ACS Synth. Biol. 2020, 9, 766–778. 10.1021/acssynbio.9b00433. 32083847

[ref35] Karig D. K.; Bessling S.; Thielen P.; Zhang S.; Wolfe J. Preservation of protein expression systems at elevated temperatures for portable therapeutic production. J. R. Soc., Interface 2017, 14, 20161039 10.1098/rsif.2016.1039. 28446704 PMC5414909

[ref36] Meister E.; Gieseler H. Freeze-dry microscopy of protein/sugar mixtures: drying behavior, interpretation of collapse temperatures and a comparison to corresponding glass transition data. J. Pharm. Sci. 2009, 98, 3072–3087. 10.1002/jps.21586. 18823013

[ref37] Jain N. K.; Roy I. Trehalose and protein stability. Curr. Protoc. Protein Sci. 2010, 59, 4.9.1–4.9.12. 10.1002/0471140864.ps0409s59. 20155732

[ref38] Kim H. C.; Kim T. W.; Park C. G.; Oh I. S.; Park K.; Kim D. M. Continuous cell-free protein synthesis using glycolytic intermediates as energy sources. J. Microbiol. Biotechnol. 2008, 18, 885–888. 18633286

[ref39] Caschera F.; Noireaux V. Synthesis of 2.3 mg/ml of protein with an all Escherichia coli cell-free transcription-translation system. Biochimie 2014, 99, 162–168. 10.1016/j.biochi.2013.11.025. 24326247

[ref40] Lee K.-H.; Kim D.-M. Recent advances in development of cell-free protein synthesis systems for fast and efficient production of recombinant proteins. FEMS Microbiol. Lett. 2018, 365, fny174 10.1093/femsle/fny174. 30084930

[ref41] Moore S. J.; Lai H. E.; Chee S. M.; Toh M.; Coode S.; Chengan K.; Capel P.; Corre C.; de Los Santos E. L.; Freemont P. S. A Streptomyces venezuelae Cell-Free Toolkit for Synthetic Biology. ACS Synth. Biol. 2021, 10, 402–411. 10.1021/acssynbio.0c00581. 33497199 PMC7901020

[ref42] Garenne D.; Thompson S.; Brisson A.; Khakimzhan A.; Noireaux V. The all-E. coliTXTL toolbox 3.0: new capabilities of a cell-free synthetic biology platform. Synth. Biol. 2021, 6, ysab017 10.1093/synbio/ysab017. PMC8546610 34712841

[ref43] Jewett M. C.; Swartz J. R. Rapid expression and purification of 100 nmol quantities of active protein using cell-free protein synthesis. Biotechnol. Prog. 2004, 20, 102–109. 10.1021/bp0341693. 14763830

[ref44] Kim D. M.; Swartz J. R. Prolonging cell-free protein synthesis with a novel ATP regeneration system. Biotechnol. Bioeng. 1999, 66, 180–188. 10.1002/(SICI)1097-0290(1999)66:3<180::AID-BIT6>3.0.CO;2-S. 10577472

[ref45] Levit M. N.; Abramczyk B. M.; Stock J. B.; Postel E. H. Interactions between Escherichia coli nucleoside-diphosphate kinase and DNA. J. Biol. Chem. 2002, 277, 5163–5167. 10.1074/jbc.M111170200. 11742005

[ref46] Chang L. L.; Pikal M. J. Mechanisms of protein stabilization in the solid state. J. Pharm. Sci. 2009, 98, 2886–2908. 10.1002/jps.21825. 19569054

[ref47] Sun Z. Z.; Yeung E.; Hayes C. A.; Noireaux V.; Murray R. M. Linear DNA for rapid prototyping of synthetic biological circuits in an Escherichia coli based TX-TL cell-free system. ACS Synth. Biol. 2014, 3, 387–397. 10.1021/sb400131a. 24303785

[ref48] Yim S. S.; Johns N. I.; Noireaux V.; Wang H. H. Protecting Linear DNA Templates in Cell-Free Expression Systems from Diverse Bacteria. ACS Synth. Biol. 2020, 9, 2851–2855. 10.1021/acssynbio.0c00277. 32926785

[ref49] Maranhao A.; Bhadra S.; Paik I.; Walker D.; Ellington A. An improved and readily available version of Bst DNA Polymerase for LAMP, and applications to COVID-19 diagnostics. medRxiv 2020, 2020.10.02.20203356 10.1101/2020.10.02.20203356.

[ref50] Mautner L.; Baillie C. K.; Herold H. M.; Volkwein W.; Guertler P.; Eberle U.; Ackermann N.; Sing A.; Pavlovic M.; Goerlich O.; Busch U.; Wassill L.; Huber I.; Baiker A. Rapid point-of-care detection of SARS-CoV-2 using reverse transcription loop-mediated isothermal amplification (RT-LAMP). Virol. J. 2020, 17, 160 10.1186/s12985-020-01435-6. 33087160 PMC7576985

[ref51] Bektaş A.; Covington M. F.; Aidelberg G.; Arce A.; Matute T.; Núñez I.; Walsh J.; Boutboul D.; Lindner A. B.; Federici F.; Jayaprakash A. Accessible LAMP-Enabled Rapid Test (ALERT) for detecting SARS-CoV-2. medRxiv 2021, 13, 742 10.3390/v13050742. PMC8146324 33922716

[ref52] Alekseenko A.; Barrett D.; Pareja-Sanchez Y.; Howard R. J.; Strandback E.; Ampah-Korsah H.; Rovsnik U.; Zuniga-Veliz S.; Klenov A.; Malloo J.; Ye S.; Liu X.; Reinius B.; Elsasser S. J.; Nyman T.; Sandh G.; Yin X.; Pelechano V. Direct detection of SARS-CoV-2 using non-commercial RT-LAMP reagents on heat-inactivated samples. Sci. Rep. 2021, 11, 1820 10.1038/s41598-020-80352-8. 33469065 PMC7815738

[ref53] Weber E.; Engler C.; Gruetzner R.; Werner S.; Marillonnet S. A modular cloning system for standardized assembly of multigene constructs. PLoS One 2011, 6, e16765 10.1371/journal.pone.0016765. 21364738 PMC3041749

[ref54] Zhu Zhenyu X. S.-Y. Method for cloning and expression of *Bsa*I restriction endonuclease and *Bsa*I methylase in *E. coli*. US20020106275, 2002.

[ref55] Bitinaite J.; Mitkaite G.; Dauksaite V.; Jakubauskas A.; Timinskas A.; Vaisvila R.; Lubys A.; Janulaitis A. Evolutionary relationship of Alw26I, Eco31I and *Esp*3I, restriction endonucleases that recognise overlapping sequences. Mol. Genet. Genomics 2002, 267, 664–672. 10.1007/s00438-002-0701-6. 12172806

[ref56] Bougueleret L.; Schwarzstein M.; Tsugita A.; Zabeau M. Characterization of the genes coding for the Eco RV restriction and modification system of Escherichia coli. Nucleic Acids Res. 1984, 12, 3659–3676. 10.1093/nar/12.8.3659. 6328432 PMC318777

[ref57] Wang P. H.; Fujishima K.; Berhanu S.; Kuruma Y.; Jia T. Z.; Khusnutdinova A. N.; Yakunin A. F.; McGlynn S. E. A Bifunctional Polyphosphate Kinase Driving the Regeneration of Nucleoside Triphosphate and Reconstituted Cell-Free Protein Synthesis. ACS Synth. Biol. 2020, 9, 36–42. 10.1021/acssynbio.9b00456. 31829622

[ref58] Jewett M. C.; Swartz J. R. Mimicking the Escherichia coli cytoplasmic environment activates long-lived and efficient cell-free protein synthesis. Biotechnol. Bioeng. 2004, 86, 19–26. 10.1002/bit.20026. 15007837

[ref59] Alissandratos A.; Caron K.; Loan T. D.; Hennessy J. E.; Easton C. J. ATP Recycling with Cell Lysate for Enzyme-Catalyzed Chemical Synthesis, Protein Expression and PCR. ACS Chem. Biol. 2016, 11, 3289–3293. 10.1021/acschembio.6b00838. 27978706

[ref60] Stark J. C.; Huang A.; Nguyen P. Q.; Dubner R. S.; Hsu K. J.; Ferrante T. C.; Anderson M.; Kanapskyte A.; Mucha Q.; Packett J. S.; Patel P.; Patel R.; Qaq D.; Zondor T.; Burke J.; Martinez T.; Miller-Berry A.; Puppala A.; Reichert K.; Schmid M.; Brand L.; Hill L. R.; Chellaswamy J. F.; Faheem N.; Fetherling S.; Gong E.; Gonzalzles E. M.; Granito T.; Koritsaris J.; Nguyen B.; Ottman S.; Palffy C.; Patel A.; Skweres S.; Slaton A.; Woods T.; Donghia N.; Pardee K.; Collins J. J.; Jewett M. C. BioBits Bright: A fluorescent synthetic biology education kit. Sci. Adv. 2018, 4, eaat5107 10.1126/sciadv.aat5107. 30083609 PMC6070313

[ref61] Collias D.; Marshall R.; Collins S. P.; Beisel C. L.; Noireaux V. An educational module to explore CRISPR technologies with a cell-free transcription-translation system. Synth. Biol. 2019, 4, ysz005 10.1093/synbio/ysz005. PMC7445873 32995532

[ref62] Williams L. C.; Gregorio N. E.; So B.; Kao W. Y.; Kiste A. L.; Patel P. A.; Watts K. R.; Oza J. P. The Genetic Code Kit: An Open-Source Cell-Free Platform for Biochemical and Biotechnology Education. Front. Bioeng. Biotechnol. 2020, 8, 941 10.3389/fbioe.2020.00941. 32974303 PMC7466673

[ref63] Burrington L. R.; Baryal E.; Hui K.; Lambert E.; Harding S. T.; Oza J. P. The Fold-Illuminator: A low-cost, portable, and disposable incubator-illuminator device. Synth. Syst. Biotechnol. 2021, 6, 95–101. 10.1016/j.synbio.2021.04.003. 33997359 PMC8099501

[ref64] Khambhati K.; Bhattacharjee G.; Gohil N.; Braddick D.; Kulkarni V.; Singh V. Exploring the Potential of Cell-Free Protein Synthesis for Extending the Abilities of Biological Systems. Front. Bioeng. Biotechnol. 2019, 7, 248–16. 10.3389/fbioe.2019.00248. 31681738 PMC6797904

[ref65] Shankar S.; Roy S.; Geary-Teeter A.; Martin G. M.; Ladd P. D. A low-cost and easy-to-use cell preservation reagent for 4 °C or room temperature sample storage. bioRxiv 2019, 745232 10.1101/745232.

[ref66] Silverman A. D.; Kelley-Loughnane N.; Lucks J. B.; Jewett M. C. Deconstructing Cell-Free Extract Preparation for in Vitro Activation of Transcriptional Genetic Circuitry. ACS Synth. Biol. 2019, 8, 403–414. 10.1021/acssynbio.8b00430. 30596483 PMC6584022

[ref67] Tanner N. A.; Zhang Y.; Evans T. C. Jr. Visual detection of isothermal nucleic acid amplification using pH-sensitive dyes. Biotechniques 2015, 58, 59–68. 10.2144/000114253. 25652028

[ref68] Vilkhovoy M.; Dai D.; Vadhin S.; Adhikari A.; Varner J. D. Absolute Quantification of Cell-Free Protein Synthesis Metabolism by Reversed-Phase Liquid Chromatography-Mass Spectrometry. J. Visualized Exp. 2019, 1–9. 10.3791/60329. 31710042

