# Supplementary material for: Constructing Cell-Free Expression Systems for Low-Cost Access
Source: ACS Synth Biol. 2022 Mar 8;11(3):1114–28. doi: 10.1021/acssynbio.1c00342 (PMC9098194; doi:10.1021/acssynbio.1c00342)
Supplement: Supplementary file 1 — sb1c00342_si_001.pdf [file sb1c00342_si_001.pdf]

SUPPLEMENTARY MATERIALS

Constructing cell-free expression systems for low-cost access

Fernando Guzman-Chavez[1]; Anibal Arce[2]; Abhinav Adhikari [3‡]; Sandra Vadhin [3‡;] Jose Antonio Pedroza-Garcia[4]; Chiara Gandini[5]; Jim W, Ajioka[6] ; Jenny Molloy[5]; Sobeida Sanchez-Nieto[4]; Jeffrey D. Varner[3]; Fernan Federici[2] & Jim Haseloff[1*].

[‡] These authors contributed equally

[1] Department of Plant Sciences, University of Cambridge, CB2 3EA, Cambridge, U.K.
[2] ANID – Millennium Institute for Integrative Biology (iBio), FONDAP Center for Genome Regulation, Institute for Biological and Medical Engineering, Schools of Engineering, Medicine and Biological Sciences, Pontificia Universidad Católica de Chile, Santiago 8330005, Chile.
[3] Robert Frederick Smith School of Chemical and Biomolecular Engineering, Cornell University, 14853, Ithaca, NY, USA.
[4] Department of Biochemistry, Faculty of Chemistry, National Autonomous University of Mexico (UNAM), 04510, Mexico City, Mexico.
[5] Department of Chemical Engineering and Biotechnology, University of Cambridge, CB3 0FD Cambridge, U.K.
[6] Department of Pathology, University of Cambridge, Tennis Court Road, CB2 1QP Cambridge, U.K.

* Corresponding Author
jh295@cam.ac.uk

FIGURE LEGENDS AND TABLES

Figure S1

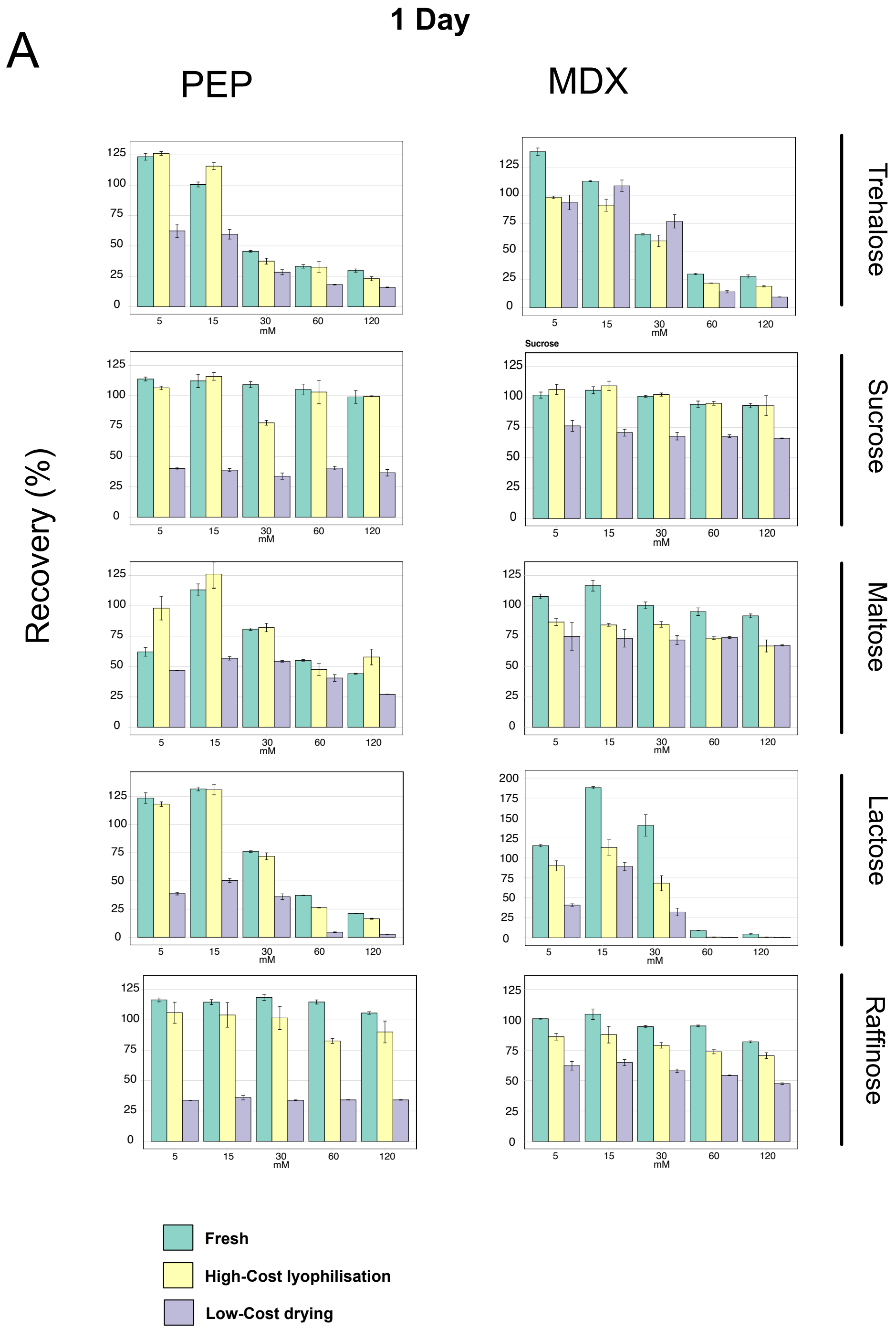

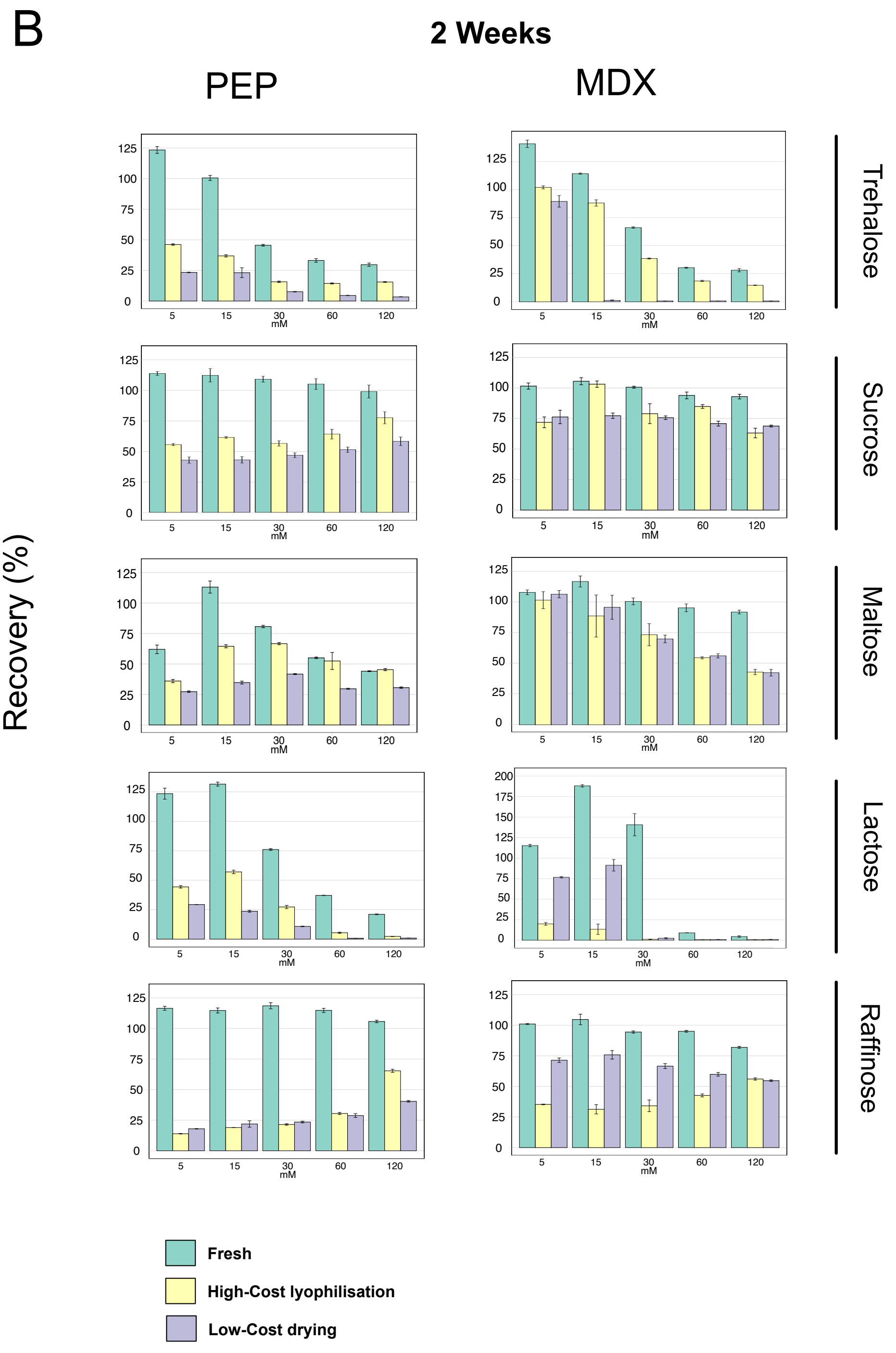

Figure S1. Lyoprotectant effects of five sugars individually added to the two cell-free formulations. Duplicate reactions, showing the effects of five sugars individually added as potential lyoprotectants to the two cell-free formulations based on PEP (A-E) and MDX (F-J) and dehydrated either by high-cost lyophilization or the low-cost drying method. Samples were dried and stored at room temperature for A) 1 day and B) 2 weeks. Cell-free reactions were rehydrated and incubated at 29°C for 15h. Plasmid psfGFP (Table S9) was used as DNA template. The final concentrations of additives in the reactions are indicated on the horizontal axes. The percentage of recovered protein production was calculated relative to that seen in fresh, additive-free reactions with the energy sources PEP or MX. Error bars represent standard deviations over three technical measurements.

Figure S2

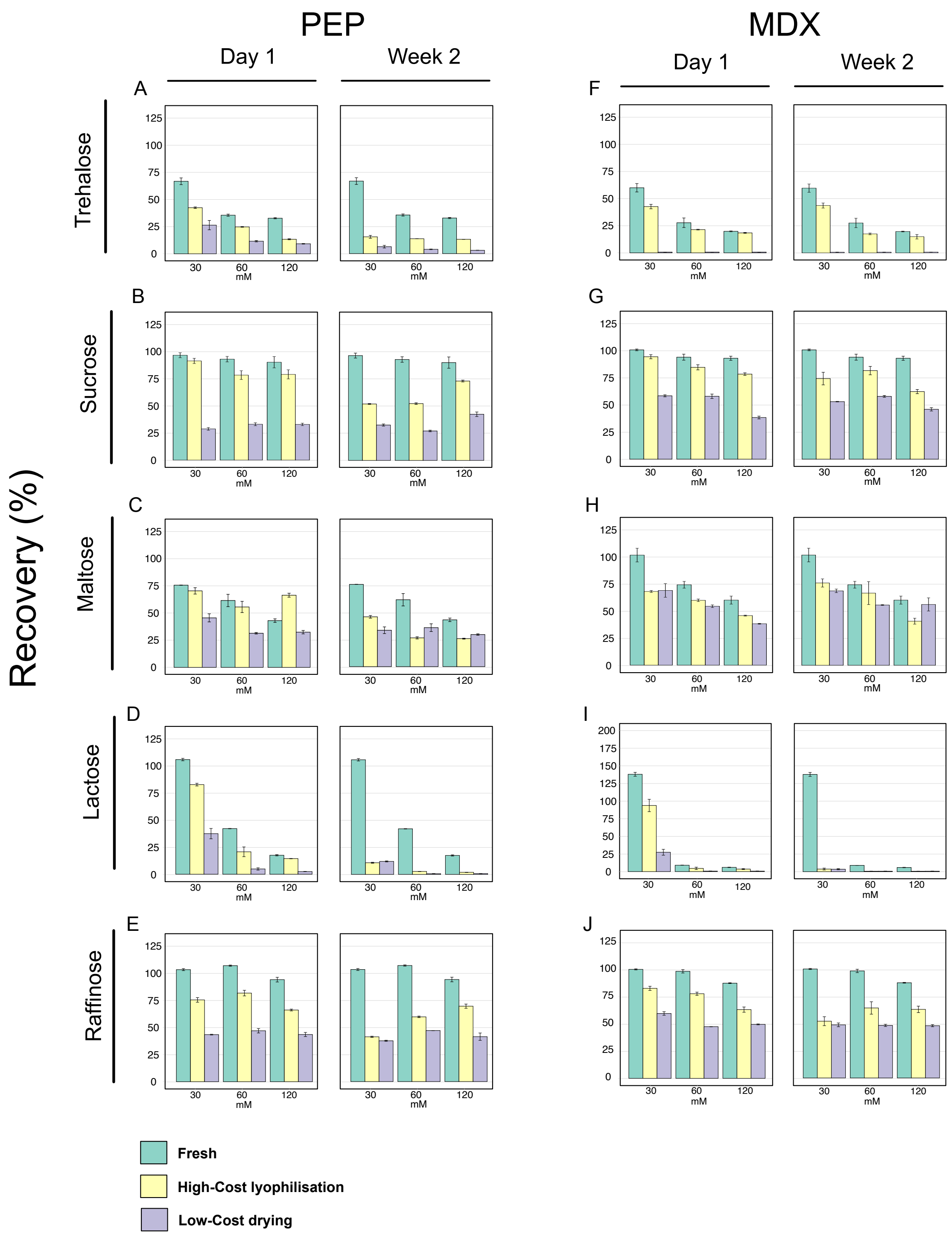

Figure S2. Lyoprotectant effects of five sugars individually added in higher concentrations. Lyoprotectant effect of five sugars individually added in two cell-free formulations based on PEP (A-E) and MDX (F-J) and dried by high-cost lyophilization or under low-cost drying. Samples were stored at room temperature for 1 day and 2 weeks. The final concentration of additives is indicated for the samples. Percentage of recovery in protein production was calculated using as 100% the RFU value from sugar free and fresh conditions with the respective energy source (PEP or MX). Cell-free reactions were incubated at 29°C for 15h. Plasmid psfGFP (Table S9) was used as DNA template. Error bars represent standard deviations over three technical measurements.

Figure S3

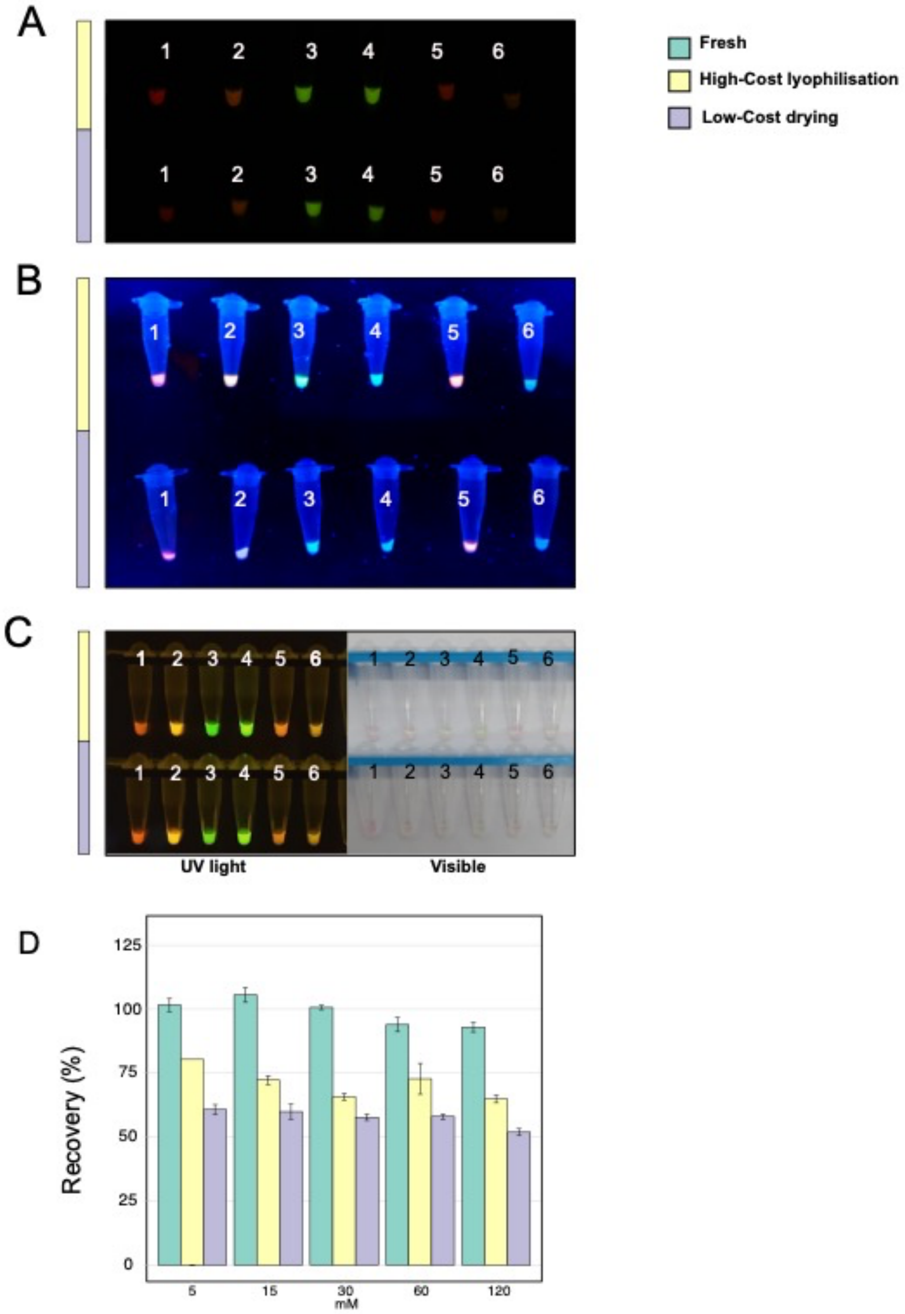

Figure S3. Sharing lyophilised and dried cell-free reactions around the globe. Fluorescent protein production after 2 weeks (A-B) and 3 months (C-D) of lyophilisation using a high and low-cost device (yellow and purple bars, respectively). A-C) Sucrose (15 mM) was added as lyoprotectant and MDX as energy source. Samples were rehydrated in A) Chile, B) Mexico and C) UK and visualized using a UV transillumінator. Fluorescent proteins produced: 1) pJL1-eforRed,2) pJL1-dTomato, 3) psfGFP, 4) pFGC-T7-RibJ-mTFP1, 5) pFGC-T7-RibJ-mScarlet, 6) pFGC-T7-RibJ-RRvT. Image presentative of three technical samples. D) Lyoprotectant effect of sucrose in the cell-free formulations based on MDX and dehydrated either by high-cost lyophilization or the low-cost drying method. Samples were stored at room temperature for 3 months. Final concentrations of sucrose in 12 µL in the lyophilised samples are indicated. Percentage of recovery in protein production was calculated using as 100% the RFU value from sugar free and fresh conditions. Cell-free reactions were incubated at 29°C for 15h. Error bars are presentative of three technical measurements.

Figure S4

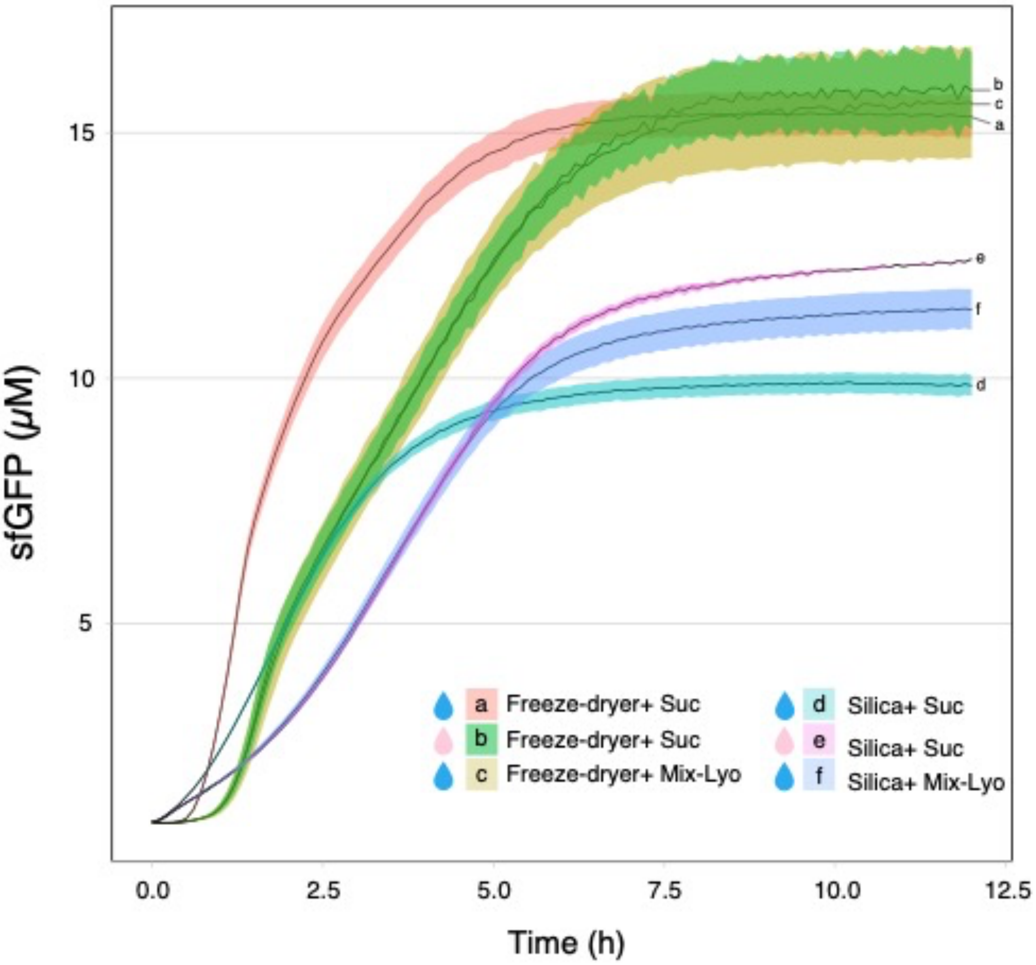

Figure S4.- Effect of lactose in cell-free reactions before and after being added during the lyophilisation/drying process. GFP production after 2 weeks of dehydration either by freeze-drying or the low-cost silica method. All the samples were tested in the cell-free formulation based on MDX.Plasmid psfGFP (Table S9) was used as DNA template. Sucrose (15 mM) or a mixture of 15 mM sucrose and 15 mM lactose (Mix-Lyo) were added as lyoprotectants. Samples were rehydrated with MQ water (blue drop) or lactose 13.7 mM (pink drop). Cell-free reactions were incubated at 29°C for 12.5 h. All error bars represent standard error over two biological replicates based on three technical measurements

Figure S5

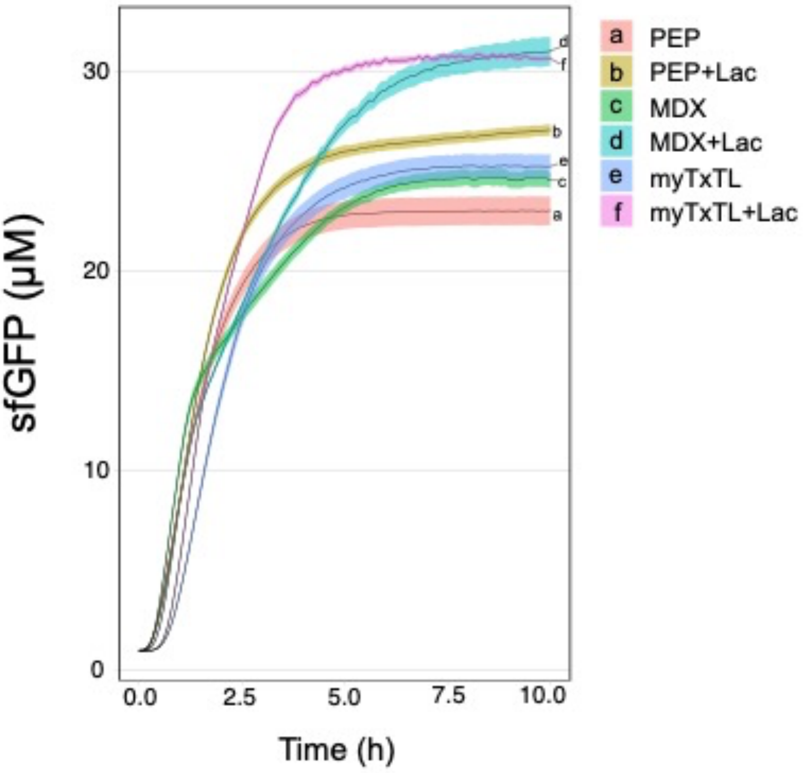

Figure S5.- Enhancer effect of lactose over sfGFP production in fresh cell-free reactions in three different formulations (PEP, MDX or commercial version). All the reactions were supplemented with 5 nM linear DNA and when was indicated 11.2 and 13.7 mM lactose was added in PEP and MDX mixture respectively. For commercial cell-free systems, 13.7 mM lactose was used. Cell-free reactions were incubated at 29°C for 10h. All error bars represent standard error over two biological replicates based on four technical measurements

Figure S6

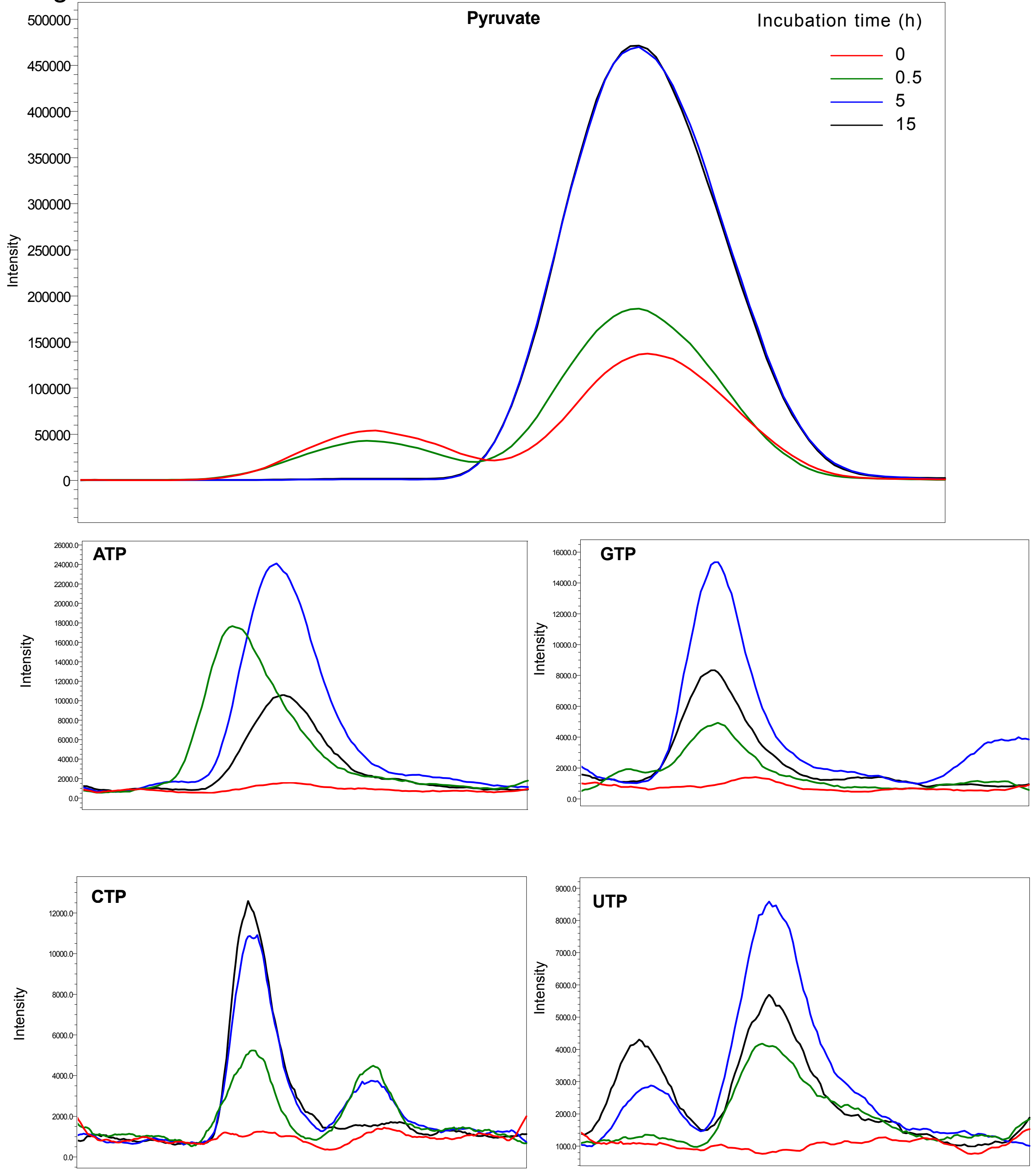

Figure S6.- Chromatograms of NTPs and pyruvate detection by LC-MS at four time points. Samples were prepared as described in table S13, replacing the indicated volume DNA with MQ water. CF presented were not supplemented with 11.2 mM lactose.

Figure S7

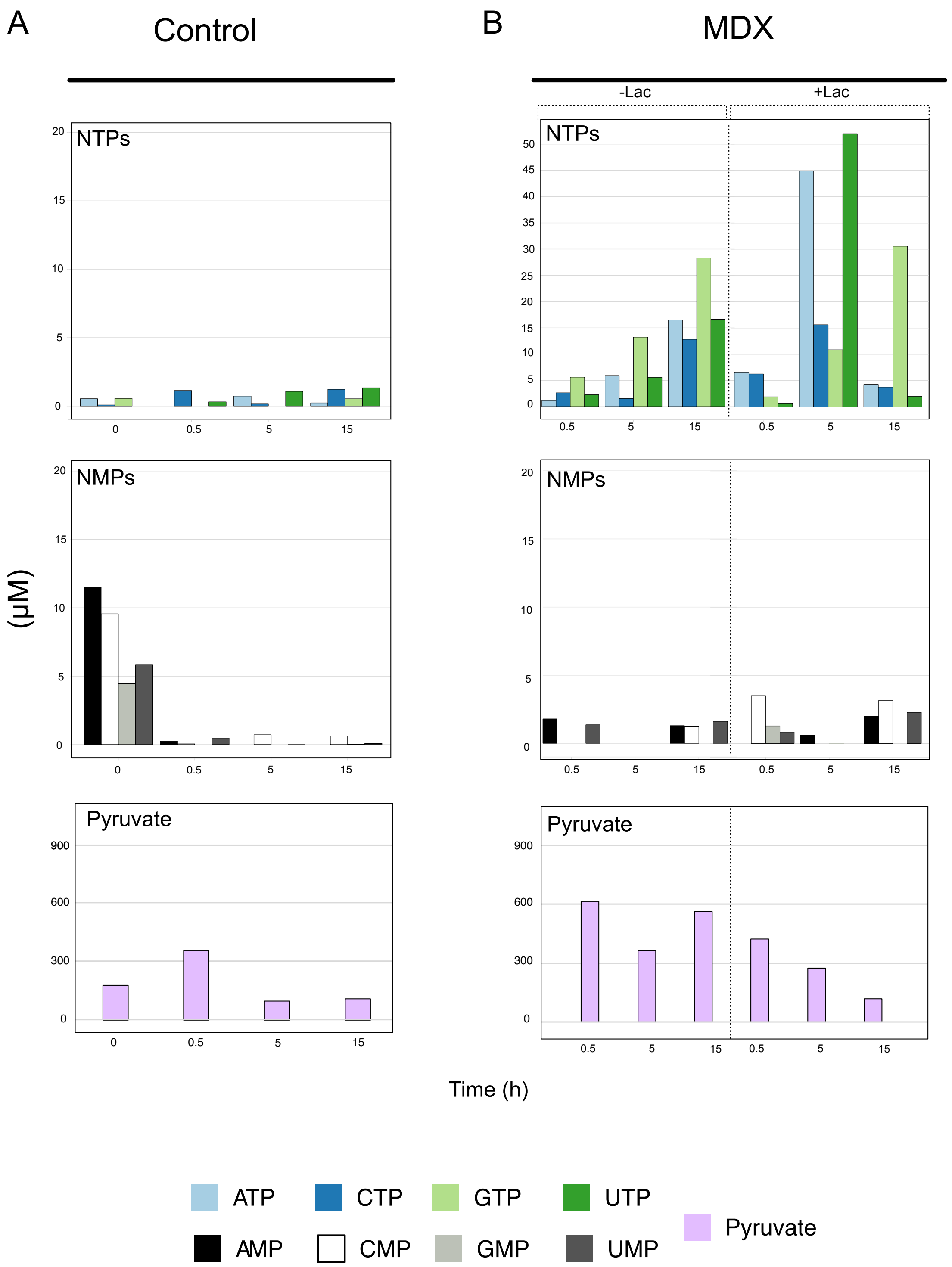

Figure S7.- Regeneration of NMPs, NTPs and pyruvate during the cell-free reactions. CFPS without A) PEP or MDX (control) and B) supplemented with MDX and lactose, measured by LC-MS at four time points. Control samples (A), which are free of any energy source as PEP or MDX, were prepared as described in Table S13, replacing PEP from the 4x ULC-Wizard mixture and DNA template with MQ water in both cases. Samples supplemented with MDX and lactose (B), were prepared as described in Table S1, keeping MDX and HEPES in the 10X energy solution according with Table S8B, variant 11. Concentrations of nucleotides and pyruvate were measured by LC-MS.

Figure S8

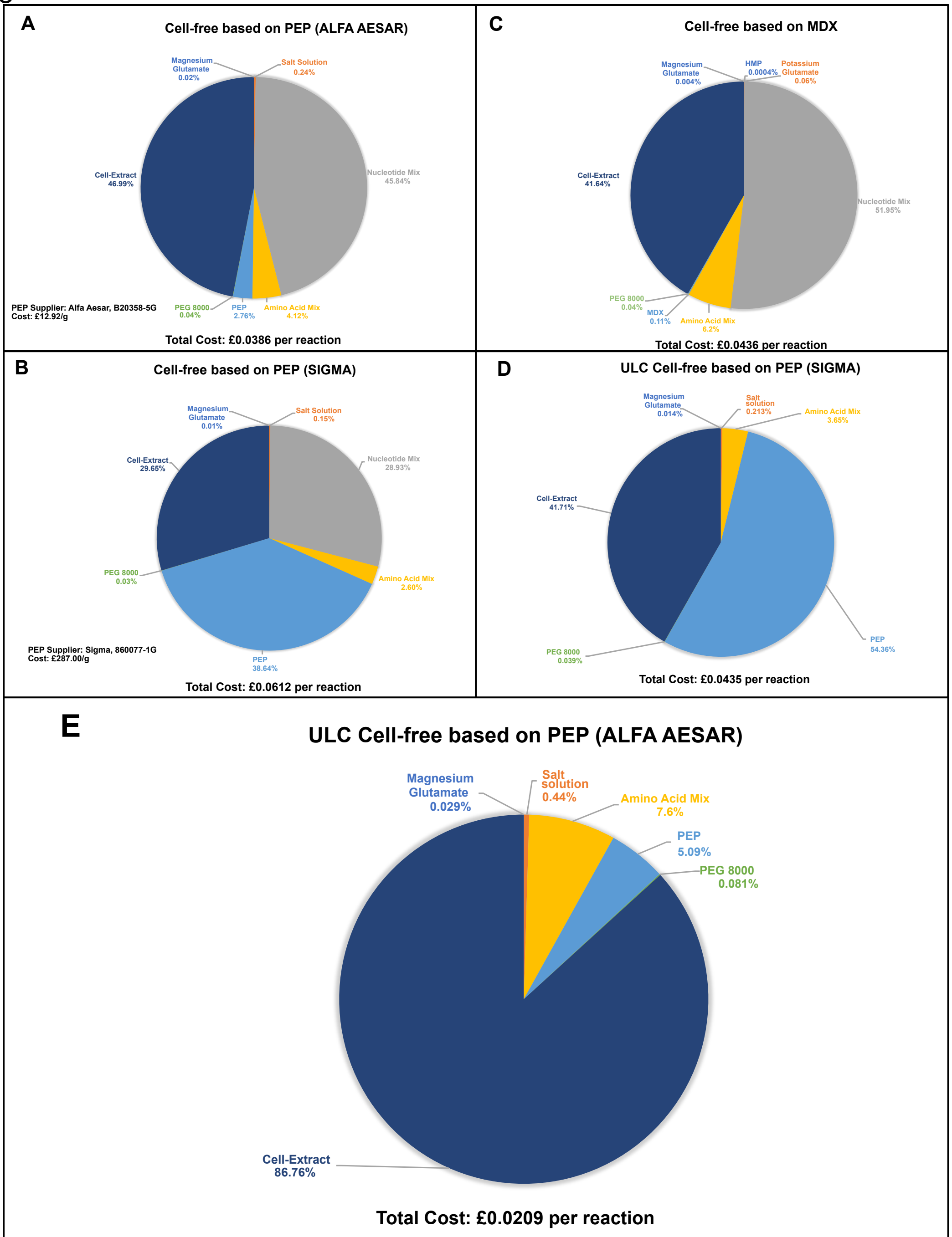

F

Formulation	Final Cost(£) per reaction	Final Cost($USD) per reaction	Fold-times regarding to MyTxTL	Fold-times regarding to PURE System	Fold-times regarding to PEP(Sigma)
MDX	0.044	0.06	203.28	656.49	1.41
PEP(Sigma)	0.061	0.09	144.49	466.62	1.00
ULC-PEP(Sigma)	0.044	0.06	203.28	656.49	1.41
PEP(Alfa Aesar)	0.039	0.05	229.09	739.82	1.59
ULC-PEP(Alfa Aesar)	0.021	0.03	425.14	1372.94	2.94
MyTxTL	8.844	12.38	1.00	3.23	0.007
PureExpress System	28.560	39.98	0.31	1.00	0.002

Figure S8.- Cost comparison between five different cell-free formulations. A) Using low-cost PEP from supplier A B) Using high-cost PEP from supplier B . C) MDX. D) ULC cell-free with PEP from supplier B. E) ULC cell-free with PEP from supplier A. F)Cost comparison versus a commercial cell-free kit (myTxTL-Linear DNA Expression Kit, Arbor 508024) and the cell-free formulation based on PEP from supplier B. Cost calculated based on a single cell-free reaction of 12 uL volume. Supplier A: Alfa Aesar. Supplier B: Sigma.

Table S1.- Energy mix composition based on **MDX for fresh samples**

Energy source: **Maltodextrin**		
Type of sample: **Fresh samples**		
	Non-sugar	Sugar
	Vol (µL)	Vol (µL)
Cell-extract	4	4
2.5X Rxn-Buffer*	4.8	4.8
**DNA (60nM)	1	1
MQ water	2.2	0.49
Sugar	n.a.	1.71
Final volume	12	12

Table S3.- Energy mix composition based on **MDX for lyophilisation mix**

Energy source: **Maltodextrin**		
Type of sample **Lyophilization mix (LM)**		
	Non-sugar	Sugar
	Vol (µL)	Vol (µL)
Cell-extract	4	4
2.5X Rxn-buffer*	4.8	4.8
**DNA	n.a.	n.a.
MQ water	2.2	0.49
Sugar	0	1.71
Final volume	**11**	**11**

Table S2.- Energy mix composition based on **PEP for fresh samples**

Energy source: **PEP**		
Type of sample **Fresh samples**		
	Non-sugar	Sugar
	Vol (µL)	Vol (µL)
Cell-extract	4	4
4X Wizard mix*	3	3
**DNA (20nM)	3	3
40% PEG-8000	0.6	0.6
MQ water	1.4	0
Sugar	0	1.4
Final volume	12	12

Table S4.- Energy mix composition based on **PEP for lyophilisation mix**

Energy source: **PEP**		
Type of sample **Lyophilization mix (LM)**		
	Non-sugar	Sugar
	Vol (µL)	Vol (µL)
Cell-extract	4	4
4X Wizard mix*	3	3
**DNA	n.a.	n.a.
40% PEG-8000	0.6	0.6
MQ water	1.4	0
Sugar	0	1.4
Final volume	**9**	**9**

* Detailed protocols for preparing all cell-free stock solutions used in this study are available at protocols.io/researchers/fernando-guzman-chavez
**Linear or circular DNA

Table S5.- Sugar concentration used in Lyophilisation Mix (LM) and fresh samples

Sugar	Stock concentration (mM)	Concentration at LM (PEP or MDX) (mM)	Concentration in cell-free reaction **PEP** (mM)	Concentration in cell-free reaction **MDX** (mM)
120	771.36	120	90.0	110.0
60	385.68	60	45.0	55.0
30	192.84	30	22.5	27.5
15	96.42	15	11.2	13.7
5	32.14	5	3.7	4.6

Table S6.- Sugars used in this study

Sugar o lyoprotectants	Cat. Number	MW(g/mol)
D-(+)-Trehalose	Merk, 1.08216.0010	342.3
D-(+)-Lactose	Sigma, 61339-25G	360.3
D-(+)-Sucrose	Fisher Scientific, S/8600/60	342.3
D-(+)-Maltose	Sigma, M5885-100G	360.3
D-(+)-Raffinose	Melford, R20500-25	594.5

Table S7.- Composition of each of the 25X nucleotide mix variants tested

A)

Reagent	Formula weight (g/mol)	Concentration in 25x nucleotide mix	Concentration in cell-free reaction	Required volume (µL) or mass (g) of precursor solution
1000 mM Putrescine	88.15	25 mM	1 mM	125 µL
1500 mM Spermidine solution	145.25	37.5 mM	1.5 mM	125 µL
50 mM NAD	663.40	8.3 mM	0.33mM	830 µL
ATP	583.36	30 mM	1.2 mM	0.0875 g
CTP	527.12	21.5 mM	0.86 mM	0.0566 g
GTP	567.1	21.5 mM	0.86 mM	0.0609 g
UTP	586.12	21.5 mM	0.86 mM	0.0630 g
CoA	767.50	6.8 mM	0.27 mM	0.0260 g
MRE600 *E.coli* tRNA	n.a.	4.3 mg/mL	170 µg/mL	215 µL
Folinic acid	511.50	0.9 mg/mL	34 µg/mL	45 µL

B)

Reagent	1	2	3	4	5	6	7	8	9	10	11
CoA	■										
tRNA	■	■									
NAD	■	■	■								
Putrescine	■	■	■	■							
Spermidine	■	■	■	■	■						
CTP	■	■	■	■	■	■					
GTP	■	■	■	■	■	■	■				
UTP	■	■	■	■	■	■	■	■			
Folinic acid	■	■	■	■	■	■	■	■	■		
ATP	■	■	■	■	■	■	■	■	■	■	

■ Indicates substrate added

TABLE S8.- Composition of each of the 10X energy solution variants tested

A)

Reagent	Formula weight (g/mol)	Concentration in 10X energy solution	Concentration in cell-free reaction	Required volume (µL) of precursor solution
HEPES pH8	238.2	510 mM	51 mM	1000
Nucleotide mix	ATP: 583.36<br>CTP: 527.12<br>GTP: 567.10<br>UTP: 586.12	15mM A,G<br>14 mM C,U	1.5 mM A,G<br>1.4 mM C,U	396
MRE600 *E.coli* tRNA	n.a.	2.02 mg/mL	202 µg/mL	160
NAD	663.4	3.39 mM	0.339mM	76.6
CoA	767.50	2.63 mM	0.263 mM	160
cAMP	329.22	7.56 mM	0.756 mM	46
Folinic acid	511.50	0.68 mM	0.068 mM	80
Spermidine	145.25	7.71 mM	0.77 mM	34
Maltodextrin	n.a.	121.44 mg/mL	12.14mg/mL	2000

B)

10 X Energy solution variants

Reagent	1	2	3	4	5	6	7	8	9	10	11
CoA	■										
tRNA	■	■									
NAD	■	■	■								
cAMP	■	■	■	■							
Spermidine	■	■	■	■	■						
CTP	■	■	■	■	■	■					
GTP	■	■	■	■	■	■	■				
UTP	■	■	■	■	■	■	■	■			
Folinic acid	■	■	■	■	■	■	■	■	■		
ATP	■	■	■	■	■	■	■	■	■	■	
Maltodextrin	■	■	■	■	■	■	■	■	■	■	■
HEPES, pH8	■	■	■	■	■	■	■	■	■	■	■

■ Indicates substrate added

Table S10.- PCR conditions using 4 primers simultaneously.

Step	Temperature (°C)	Time (sec)	Cycles
Initial denaturation	98	90	1
Denaturation	98	10	
Annealing	61	20	35
Elongation	72	60	
Final elongation	72	120	1
Storage	4	n.a.	n.a.

Table S9.- Plasmids used in this study.

Name	Description	Marker	Reference
psfGFP	pT7-RiboJ-sfGFP-T7	TetR	Arce et al., 2021 [1]
pFGC-T7-RJBB*	pT7-RiboJ-LacZ⟨-T7	KanR	This study
pFGC-T7RibJ-mTFP1	pT7-RiboJ-mTFP1-T7	KanR	This study
pFGC-T7RibJ-RRvT	pT7-RiboJ-RRvT-T7	KanR	This study
pFGC-T7RibJ-mScarlet	pT7-RiboJ-mScarlet-T7	KanR	This study
pJL1-dTomato	pT7-dTomato-T7	KanR	Stark, et al., 2018[2] Addgene:102631
pJL1-eforRed	pT7-eforRed-T7	KanR	Huang, et al., 2018[3] Addgene:106320
pKAR2-Br512	pT7-8XHisTag-Br512-tetPA	AmpR	Mautner et al., 2020 [4] Addgene: 161875
iluxpGEX	Lux operon	AmpR	Gregor, et al.,2018 [5]

* Acceptor or backbone plasmid

Table S11.- Primers used in this study.

Name	Sequence 5'->3'	Template	Use	Reference
U1F	CATTACTCGCATCCATTCTCAGGCTGTCTCGTCTCGTCTC	pSfGFP_AA4	Linear DNA-Long Flanks	Arce et al., 2021 [1]
UXR	GGTGGAAGGGCTCGGAGTTGTGGTAATCTATGTATCCTGG	pSfGFP_AA4	Linear DNA-Long Flanks	Arce et al., 2021 [1]
336	AATTAATACGACTCACTATAGGGAGCTG	pSfGFP_AA4	Linear DNA-Short Flanks	This study
231	CAGCAAAAAACCCCTCAAGACCCGTTTAGAGGC	pSfGFP_AA4	Linear DNA-Short Flanks	This study
292_pT7_RiboJ-FW (Adapter oligo)	CATTACTCGCATCCATTCTCAGGCTGTCTCGTCTCGTCTCCGGAAGACATGCTTAGGAGCCTGCATTAGGATCGATCTCGATCCCGCGAAATTAATACGACTCACTATAGGGAGCTGTCACCGGATGTGCTTTCCGGTCTGATGAGTCCGTGAGGACGAAACAGCCTCTACAAATAATTTTGTTTAATACTAGACAGAAA	BsaI gBlock	4-Oligo PCR	This study
293_TermT7_Rw (Adapter oligo)	GGGTGGAAGGGCTCGGAGTTGTGGTAATCTATGTATCCTGGCCGCGCGCGGCTTGGATTCTGCGTTTGTTTCCGTCTACGAACTCCCAGCCTGAAGACATGACAAAGCGAGGTTTTCAGCAAAAAACCCCTCAAGACCCGTTTAGAGGCCCCAAGGGGTTATGCTAGTTATTGCTCAGCGGCCTAGGCGACCT	BsaI gBlock	4-Oligo PCR	This study
298_BsaI_Core-Fw	acgaaacagcctctacaaataattttgtttaatactagacagaaacagaggagatatgcaATGGGGAAAAAGGCCGAATA	BsaI gBlock	4-Oligo PCR	This study
299_BsaI_Core-Rv	atgctagttattgctcagcggcctaggcgacctTCAATCCAGATCGGCAAAG	BsaI gBlock	4-Oligo PCR	This study
Core-Fw Backbone	acgaaacagcctctacaaataattttgtttaatactagacagaaacagaggagatatgca**ATG-N**$_{15\text{-}23}$	Any template	4-Oligo PCR	This study
Core-Rv Backbone	atgctagttattgctcagcggcctaggcgacct-**N**$_{18\text{-}23}$ (Stop codon must be included)	Any template	4-Oligo PCR	This study
272_ACTB-F3	AGTACCCCATCGAGCACG	ActinB gBlock	LAMP assay	SARS-CoV-2 Rapid Colorimetric LAMP Assay Kit NEB #E2019S
273_ACTB-B3	AGCCTGGATAGCAACGTACA	ActinB gBlock	LAMP assay	
274_ACTB-FIP	GAGCCACACGCAGCTCATTGTATCACCAACTGGGACGACA	ActinB gBlock	LAMP assay	
275_ACTB-BIP	CTGAACCCCAAGGCCAACCGGCTGGGGTGTTGAAGGTC	ActinB gBlock	LAMP assay	

276_ACTB-LF	TGTGGTGCCAGATTTTCTCCA	ActinB gBlock	LAMP assay	
277_ACTB-LB	CGAGAAGATGACCCAGATCATGT	ActinB gBlock	LAMP assay	

Table S12.- Sequences used in this study

Name	Sequence (5'->3').	Reference
**BsaI**	ATGGGGAAAAAGGCCGAATATGGACAGGGTCATC<br>CTATCTTCCTTGAGTACGCTGAACAGATCATTCAA<br>CACAAGGAGTACCAGGGTATGCCAGATCTGCGTT<br>ATCCGGATGGGCGTATTCAGTGGGAGGCACCTTC<br>TAACCGTAAATCGGGCATCTTTAAAGACACCAACA<br>TCAAGCGTCGTAAATGGTGGGAGCAAAAAGCGAT<br>CTCCATTGGAATCGACCCTTCTTCGAATCAGTGGA<br>TCTCCAAGACAGCGAAATTAATCCACCCGACAAT<br>GCGTAAACCCTGTAAGAAGTGTGGACGTATTATG<br>GATCTTCGCTACTCGTATCCCACAAAAAACCTGAT<br>CAAGCGCATCCGTAAGTTACCATATGTCGACGAA<br>TCTTTTGAAATCGACTCACTGGAGCATATTCTGAA<br>GCTGATCAAACGCTTGGTATTACAATATGGGGAC<br>AAAGTTTATGACGATTTACCCAAGCTGTTAACTTG<br>TAAAGCGGTTAAAAACATTCCTCGTTTGGGAAATG<br>ATCTGGACACGTGGTTAAACTGGATTGACTCCGT<br>CTATATCCCTAGCGAACCATCAATGCTGTCACCG<br>GGAGCTATGGCTAATCCACCAGATCGCTTGGACG<br>GGTTCCACTCCCTTAATGAGTGCTGTCGTAGTCAT<br>GCGGATCGTGGCCGCTGGGAAAAGAATCTTCGCT<br>CTTATACAACTGATCGTCGCGCATTCGAATACTGG<br>GTCGATGGAGACTGGGTAGCGGCTGATAAATTAA<br>TGGGACTTATCCGTACCAATGAGCAAATCAAGAA<br>GGAAACATGTTTAAACGATAACCACCCTGGTCCTT<br>GCAGTGCCGATCATATCGGTCCGATCTCTCTGGG<br>ATTTGTCCATCGTCCTGAATTTCAACTGCTTTGTA<br>ACTCCTGTAATTCTGCAAAGAACAACCGTATGACT<br>TTCAGCGACGTTCAGCATCTTATCAACGCCGAAAA<br>TAATGGCGAAGAGGTCGCCAGTTGGTACTGTAAA<br>CATATCTGGGACTTACGCAAACATGACGTAAAGAA<br>CAATGAAAATGCGTTACGCCTTAGTAAGATCCTTC<br>GTGACAACCGTCACACTGCGATGTTCATTCTTAGT<br>GAGCTTTTGAAAGACAATCATTATCTGTTCCTTTC<br>AACGTTTTTAGGCCTTCAATATGCAGAGCGTTCAG<br>TGTCCTTTTCTAACATCAAGATTGAAAATCACATCA<br>TTACTGGGCAGATCTCGGAACAACCCCGTGACAC<br>TAAATATACAGAAGAACAAAAAGCTCGCCGCATG<br>CGCATCGGCTTTGAAGCCCTTAAGAGTTACATCG<br>AAAAGGAGAACCGCAACGCCCTTTTGGTGATCAA<br>TGATAAGATCATCGACAAAATCAATGAAATCAAGA<br>ACATCCTTCAGGACATTCCCGATGAATACAAGTTA<br>TTAAACGAGAAAATCAGTGAGCAATTCAATAGCGA<br>GGAAGTCTCTGATGAATTGTTGCGTGATTTGGTTA<br>CACACCTGCCTACGAAGGAATCAGAGCCAGCAAA	GenBank: AY453694.1

	CTTTAAGCTGGCGCGCAAGTATTTACAAGAGATCATGGAAATCGTAGGGGACGAACTGTCCAAGATGTGGGAGGACGAACGCTATGTTCGCCAGACCTTTGCCGATCTGGATTGA	
**ActinB**	TTCCTATGTGGGCGACGAGGCCCAGAGCAAGAGAGGCATCCTCACCCTGAAGTACCCCATCGAGCACGGCATCGTCACCAACTGGGACGACATGGAGAAAATCTGGCACCACACCTTCTACAATGAGCTGCGTGTGGCTCCCGAGGAGCACCCCGTGCTGCTGACCGAGGCCCCCCTGAACCCCAAGGCCAACCGCGAGAAGATGACCCAGATCATGTTTGAGACCTTCAACACCCCAGCCATGTACGTTGCTATCCAGGCTGTGCTATCCCTGTACGCCTCTGGCCGTACCACTGGCATCGTGATGGACTC	NCBI Reference Sequence: NM_001101.5
**mTFP**	ATGGTGAGCAAGGGCGAGGAAACCACAATGGGCGTAATCAAGCCCGACATGAAGATCAAGCTGAAGATGGAGGGCAACGTGAATGGCCACGCCTTCGTGATCGAGGGCGAGGGCGAGGGCAAGCCCTACGACGGCACCAACACCATCAACCTGGAGGTGAAGGAGGGAGCCCCCCTGCCCTTCTCCTACGACATTCTGACCACCGCGTTCGCCTACGGCAACAGGGCCTTCACCAAGTACCCCGACGACATCCCCAACTACTTCAAGCAGTCCTTCCCCGAGGGCTACTCTTGGGAGCGCACCATGACCTTCGAGGACAAGGGCATCGTGAAGGTGAAGTCCGACATCTCCATGGAGGAGGACTCCTTCATCTACGAGATACACCTCAAGGGCGAGAACTTCCCCCCCAACGGCCCCGTGATGCAGAAGAAAACCACCGGCTGGGACGCCTCCACCGAGAGGATGTACGTGCGCGACGGCGTGCTGAAGGGCGACGTCAAGCACAAGCTGCTGCTGGAGGGCGGCGGCCACCACCGCGTTGACTTCAAGACCATCTACAGGGCCAAGAAGGCGGTGAAGCTGCCCGACTATCACTTTGTGGACCACCGCATCGAGATCCTGAACCACGACAAGGACTACAACAAGGTGACCGTTTACGAGAGCGCCGTGGCCCGCAACTCCACCGACGGCATGGACGAGCTGTACAAGTAA	Ai, et al., 2006 [6]
**RRvT**	ATGGTGAGCAAGGGCGAGGAGGTGATCAAGGAATTCATGAGGTTCAAGGTGAGGATGGAGGGCTCCATGAATGGACATGAGTTTGAAATTGAAGGAGAGGGAGAGGGACGCCCTTATGAAGGCACCCAGACCGCCAAGCTGAAGGTGACCAAGGGCGGCCCCCTGCCCTTCGCCTGGGACATTCTGTCCCCTCAGTTTATGTATGGATCTAAGGCTTATGTCAAACATCCTGCTGATATTCCCGACTACAAGAAGCTGAGCTTCCCCGAGGGCTTCAAGTGGGAGAGGGTTATGAACTTCGAAGATGGAGGACTGGTCACAGTCACACAGGATTCCTCCCTGCAGGATGGAACACTGATTTACAATGTGAAGATGAGGGGCACCAACTTTCCACCCGACGGCCCCGTGATGCAAAAGAAAACAATGGGATGGGAGGCTTCCACAGAACGCCTGTATCCTCGTGATGGAGTCCTGAAAGGAGAGATCCACCAGGCCCTGAAGCTGAAGGACGGCGGCCACTACCTGGTGGAGTTTAAGACCAT	Wiens et al., 2016 [7]

	TTATATGGCTAAAAAACCTGTCCAACTGCCTGGAT ATTATTATGTCGATACAAAACTGGACATCACCAGC CACAACGAGGACTACACCATCGTGGAGCAGTACG AGAGGAGCGAGGGCCGCCATCATCTGTTCCTCTA TGGAATGGATGAACTCTATAAAGGCAGCACCGGC AGCGGCAGCTCCGGCCCCATGGTTTCCAAAGGA GAAGAAGCCATTAAAGAGTTTATGCGCTTCAAAGT CAGCATGGAAGGCAGCATGAACGGCCACGAGTT CGAGATCGAGGGCGAGGGCGAGGGCAGGCCCTA CGAGGGAACACAGACAGCTAAACTGAAAGTCACA AAAGGAGGACCTCTGCCTTTCGCTTGGGATATCC TGAGCCCCCAGTTCATGTACGGCAGCAAGGCCTA CGTGAAGCACCCCGCCGACATCCCTGATTATAAA AAACTGTCCTTTCCTGAAGGATTCAGATGGGAAC GCGTCATGAATTTCGAGGACGGCGGCCTGGTGA CCGTGACCCAGGACAGCAGCATCCAGGACGGCA CCCTGATCTATAAAGTCAAAGTGCGCGGAACAAA TTTCCCTCCTGATGGACCTGTCATGCAGAAAAAA CCATGGGCTGGGAAGCCAGCACCGAGAGGCTGT ACCCCAGGGACGGCGTGCTGAAGGGCGAAATTC ATCAGGCTCTGAAACTGAAAGATGGAGGACATTA TCTGGTCGAATTCAAAACAATCTACATGGCCAAGA AGCCCGTGCAGCTCCCCGGCTACTACTACGTGGA CACCAAGCTGGATATTACATCCCATAATGAAGATT ATACAGTTGTCGAACAGTATGAACGCTCCGAAGG AAGGCACCACCTCTTTCTGTACGGCATGGACGAG CTGTACAAGTAA	
**mScarlet**	ATGGTGAGCAAGGGCGAGGCAGTGATCAAGGAG TTCATGCGGTTCAAGGTGCACATGGAGGGCTCCA TGAACGGCCACGAGTTCGAGATCGAGGGCGAGG GCGAGGGCCGCCCCTACGAGGGCACCCAGACCG CCAAGCTGAAGGTGACCAAGGGTGGCCCCCTGC CCTTCTCCTGGGACATCCTGTCCCCTCAGTTCAT GTACGGCTCCAGGGCCTTCATCAAGCACCCCGCC GACATCCCCGACTACTATAAGCAGTCCTTCCCCG AGGGCTTCAAGTGGGAGCGCGTGATGAACTTCGA GGACGGCGGCGCCGTGACCGTGACCCAGGACAC CTCCCTGGAGGACGGCACCCTGATCTACAAGGTG AAGCTCCGCGGCACCAACTTCCCTCCTGACGGCC CCGTAATGCAGAAGAAGACAATGGGCTGGGAAGC GTCCACCGAGCGGTTGTACCCCGAGGACGGCGT GCTGAAGGGCGACATTAAGATGGCCCTGCGCCT GAAGGACGGCGGCCGCTACCTGGCGGACTTCAA GACCACCTACAAGGCCAAGAAGCCCGTGCAGATG CCCGGCGCCTACAACGTCGACCGCAAGTTGGAC ATCACCTCCCACAACGAGGACTACACCGTGGTGG AACAGTACGAACGCTCCGAGGGCCGCCACTCCA CCGGCGGCATGGACGAGCTGTACAAGTAA	Bindels, et al., 2017 [8]

Table S13.- Ultra-low-cost (ULC) cell-free formulation based on **PEP for fresh samples**

A) **ULC-PEP** cell-free reaction

**Precursor solution**	**Required quantity (µL)**
1) 96.4 mM lactose	1.4
2) 40% PEG-8000	0.6
3) DNA (20 nM)	3
4) 4x ULC- Wizard mix	3
5) Cell extract	4
Final volume	12

B) 4X **ULC-PEP** Wizard mix

**Precursor solution**	**Required quantity (µL)**
1) Autoclaved MQ water	120
2) 1000mM magnesium glutamate	20*
3) 10x Salt solution mix**	200
4) 25x 19 Amino acid mix**	80
5) 25x PEP**	80
Final volume	500

* For a final concentration of 10 mM in ULC-cell-free reaction.
**Detailed protocols for preparing all cell-free stock solutions used in this study are available at protocols.io/researchers/fernando-guzman-chavez

REFERENCES

[1] Arce, A., Guzman Chavez, F., Gandini, C., Puig, J., Matute, T., Haseloff, J., Dalchau, N., Molloy, J., Pardee, K., and Federici, F. (2021) Decentralizing Cell-Free RNA Sensing With the Use of Low-Cost Cell Extracts, *Frontiers in Bioengineering and Biotechnology 9*, 1-11.
[2] Stark, J. C., Huang, A., Nguyen, P. Q., Dubner, R. S., Hsu, K. J., Ferrante, T. C., Anderson, M., Kanapskyte, A., Mucha, Q., Packett, J. S., Patel, P., Patel, R., Qaq, D., Zondor, T., Burke, J., Martinez, T., Miller-Berry, A., Puppala, A., Reichert, K., Schmid, M., Brand, L., Hill, L. R., Chellaswamy, J. F., Faheem, N., Fetherling, S., Gong, E., Gonzalzles, E. M., Granito, T., Koritsaris, J., Nguyen, B., Ottman, S., Palffy, C., Patel, A., Skweres, S., Slaton, A., Woods, T., Donghia, N., Pardee, K., Collins, J. J., and Jewett, M. C. (2018) BioBits Bright: A fluorescent synthetic biology education kit, *Sci Adv 4*, eaat5107.
[3] Huang, A., Nguyen, P. Q., Stark, J. C., Takahashi, M. K., Donghia, N., Ferrante, T., Dy, A. J., Hsu, K. J., Dubner, R. S., Pardee, K., Jewett, M. C., and Collins, J. J. (2018) BioBits Explorer: A modular synthetic biology education kit, *Sci Adv 4*, eaat5105.
[4] Mautner, L., Baillie, C. K., Herold, H. M., Volkwein, W., Guertler, P., Eberle, U., Ackermann, N., Sing, A., Pavlovic, M., Goerlich, O., Busch, U., Wassill, L., Huber, I., and Baiker, A.

(2020) Rapid point-of-care detection of SARS-CoV-2 using reverse transcription loop-mediated isothermal amplification (RT-LAMP), *Virol J 17*, 1-14.
[5] Gregor, C., Gwosch, K. C., Sahl, S. J., and Hell, S. W. (2018) Strongly enhanced bacterial bioluminescence with the ilux operon for single-cell imaging, *Proc Natl Acad Sci U S A 115*, 962-967.
[6] Ai, H. W., Henderson, J. N., Remington, S. J., and Campbell, R. E. (2006) Directed evolution of a monomeric, bright and photostable version of Clavularia cyan fluorescent protein: structural characterization and applications in fluorescence imaging, *Biochem J 400*, 531-540.
[7] Wiens, M. D., Shen, Y., Li, X., Salem, M. A., Smisdom, N., Zhang, W., Brown, A., and Campbell, R. E. (2016) A Tandem Green-Red Heterodimeric Fluorescent Protein with High FRET Efficiency, *Chembiochem 17*, 2361-2367.
[8] Bindels, D. S., Haarbosch, L., van Weeren, L., Postma, M., Wiese, K. E., Mastop, M., Aumonier, S., Gotthard, G., Royant, A., Hink, M. A., and Gadella, T. W., Jr. (2017) mScarlet: a bright monomeric red fluorescent protein for cellular imaging, *Nat Methods 14*, 53-56.
